# Cross-utilisation of template RNAs by alphavirus replicases

**DOI:** 10.1371/journal.ppat.1008825

**Published:** 2020-09-04

**Authors:** Laura Sandra Lello, Age Utt, Koen Bartholomeeusen, Sainan Wang, Kai Rausalu, Catherine Kendall, Sandra Coppens, Rennos Fragkoudis, Andrew Tuplin, Luke Alphey, Kevin K. Ariën, Andres Merits

**Affiliations:** 1 Institute of Technology, University of Tartu, Tartu, Estonia; 2 Department of Biomedical Sciences, Institute of Tropical Medicine, Antwerpen, Belgium; 3 Faculty of Biological Sciences and Astbury Centre for Structural and Molecular Biology, University of Leeds, Leeds, United Kingdom; 4 University of Nottingham, School of Veterinary Medicine and Science, Loughborough, United Kingdom; 5 The Pirbright Institute, Woking, United Kingdom; 6 Department of Biomedical Sciences, University of Antwerp, Antwerpen, Belgium; University of Colorado Denver, UNITED STATES

## Abstract

Most alphaviruses (family *Togaviridae*) including Sindbis virus (SINV) and other human pathogens, are transmitted by arthropods. The first open reading frame in their positive strand RNA genome encodes for the non-structural polyprotein, a precursor to four separate subunits of the replicase. The replicase interacts with *cis*-acting elements located near the intergenic region and at the ends of the viral RNA genome. A *trans*-replication assay was developed and used to analyse the template requirements for nine alphavirus replicases. Replicases of alphaviruses of the Semliki Forest virus complex were able to cross-utilize each other’s templates as well as those of outgroup alphaviruses. Templates of outgroup alphaviruses, including SINV and the mosquito-specific Eilat virus, were promiscuous; in contrast, their replicases displayed a limited capacity to use heterologous templates, especially in mosquito cells. The determinants important for efficient replication of template RNA were mapped to the 5' region of the genome. For SINV these include the extreme 5'- end of the genome and sequences corresponding to the first stem-loop structure in the 5' untranslated region. Mutations introduced in these elements drastically reduced infectivity of recombinant SINV genomes. The *trans*-replicase tools and approaches developed here can be instrumental in studying alphavirus recombination and evolution, but can also be applied to study other viruses such as picornaviruses, flaviviruses and coronaviruses.

## Author summary

Alphaviruses are positive-strand RNA viruses, most of which use mosquitoes to spread between vertebrate hosts; many are human pathogens with potentially severe medical consequences. Some alphavirus species are believed to have resulted from the recombination between different members of the genus and there is evidence of movement of alphaviruses between continents. Here, a novel assay uncoupling viral replicase and template RNA production was developed and used to analyse cross-utilization of alphavirus template RNAs. We observed that replicases of closely related alphaviruses belonging to the Semliki Forest virus complex can generally use each other’s template RNAs as well as those of distantly related outgroup viruses. In contrast, replicases of outgroup viruses clearly preferred homologous template RNAs. These trends were observed in both mammalian and mosquito cells, with template preferences generally more pronounced in mosquito cells. Interestingly, the template RNA of the mosquito-specific Eilat virus was efficiently used by other alphavirus replicases while Eilat replicase could not use heterologous templates. Determinants for template selectivity were mapped to the beginning of the RNA genome and template recognition was more likely based on the recognition of RNA sequences than recognition of structural elements formed by the RNAs.

## Introduction

The genus *Alphavirus* (family *Togaviridae*) comprises approximately 30 known virus species. Many of these are “arboviruses”, infecting vertebrate hosts and are transmitted through the bite of an arthropod vector, commonly mosquitoes. Many alphaviruses present a threat to human health. These include chikungunya virus (CHIKV), which recently (re-)emerged in Asia, Africa and America [1], o’nyong-nyong virus (ONNV) that is widespread in Africa [2], Ross River virus (RRV) which is epidemic in Australia/Oceania [3] and Venezuelan equine encephalitis virus (VEEV) which is found in the Americas [4,5]. In addition to arboviruses there are also horizontally transmitted alphaviruses such as salmon pancreas disease virus (salmonid alphavirus, SAV) infecting aquatic species. Some alphaviruses, such as Eilat virus (EILV), lack a vertebrate host and infect arthropods exclusively [6,7].

The members of the genus *Alphavirus* are divided into several groups (complexes) that form three major clades [6]. Alphaviruses having vertebrate hosts are also often divided according to their geographical distribution and pathogenesis associated with their infection. New World alphaviruses, exemplified by VEEV and Western equine encephalitis virus (WEEV), cause encephalitis while Old World alphaviruses, including Sindbis virus (SINV, type member of the genus), CHIKV, ONNV, RRV and Barmah Forest virus (BFV) cause fever, rash and arthritic symptoms. This division is supported by molecular biological evidence indicating that Old and New World alphaviruses have different mechanisms to suppress host cell transcription and to counteract host antiviral mechanisms [8]. There are also notable differences in the host factors these viruses require for their genome replication [9]. However, the correlation between current geographical distributions and categorization as New or Old World alphaviruses is not absolute. For example, Mayaro virus (MAYV) found in South America belongs to the Semliki Forest virus (SFV) complex of Old World alphaviruses [6]. Similarly, phylogenetic analysis suggests that SINV has its origin in the New World regions [10]. Thus, it is likely that alphaviruses have spread from one geographical area to another, possibly in migratory birds [10,11]. WEEV has its ancestry in a recombination event between Eastern equine encephalitis virus and SINV-like viruses [12]. Taken together, the evolutionary history of alphaviruses is complex and only partially understood.

Alphaviruses have a positive strand RNA genome of approximately 12 kb in length, with a 5' type-0 N 7-methylguanosine cap structure, a 3' poly(A) tail and contain two open reading frames (ORF). The first ORF encodes four non-structural proteins (nsP1-4) as polyprotein precursors P1234 or P123, with P1234 being synthesized by read-through of a stop codon between nsP3 and nsP4 [13]. These polyproteins are processed by the protease activity of the nsP2 region into intermediates and finally individual nsPs. The proteolytic processing, leading to the formation of a functional replicase complex, is tightly regulated. First, P1234 is processed into P123 and nsP4. This results in formation of the early replicase (P123+nsP4) catalysing the synthesis of negative strand RNA [14]. These events coincide with the formation of membrane bound replicase complexes termed spherules [15,16]. P123 is subsequently cleaved into nsP1 and P23. This is a delayed and precisely timed event; an acceleration of P123 processing attenuates viral replication or completely blocks infectivity [17]. The cleavage of P123 is rapidly followed by *trans*-cleavage of P23 into mature nsP2 and nsP3 which, together with nsP1 and nsP4 form the late replicase which is responsible for the synthesis of new genomic RNA as well as sub-genomic (SG) RNA, corresponding to the 3’ one third of the virus genome and used as the mRNA for expression of structural proteins [14,18]. The late replicase is very active and the copy numbers of new positive strand RNAs can reach hundreds of thousands per cell. In addition to viral nsPs and cellular membranes, replicase formation and functioning requires the recruitment of various host proteins. It has been shown that some of these proteins are absolutely necessary for only one/a few alphaviruses [19] while others are essential for larger groups of alphaviruses [9].

The alphavirus genomes contain three untranslated regions (UTRs), all of which play crucial roles in virus infection [20]. The 5' UTR is less than 100nt in length while the 3' UTR is up to 900nt in length. The length of the 3' UTR varies significantly between viruses and even between different isolates of the same virus. Most of this variation is due to different copy numbers of repeated sequence motifs. For CHIKV the presence of repeated motifs is essential for efficient replication in mosquito cells but has little, if any, impact on replication in vertebrate cells [21–23]. The 3' UTR interacts with host protein HuR increasing the stability of viral RNAs and promoting infection both in vertebrate and mosquito cells [24]. A third, intragenic, non-coding region, typically of around 50nt, is located between the two ORFs. There are four conserved sequence elements (cse) in the alphavirus genome. The first cse is located at the very 5' end of the genome and the second, so called 51-nt cse, is located in the nsP1 encoding region. The third 21-nt cse mostly overlaps with the region encoding the C-terminus of nsP4, in the downstream region of the first ORF. The final 19-nt cse, is located immediately upstream of the poly(A) tail [25]. The basic significance of these elements is reasonably well-understood. The two 5' elements and 3' cse function in a coordinated manner in the synthesis of the negative and positive strands of the virus genomes [26]. The element located within the nsP4 encoding region functions as a SG promoter [27]. It has been demonstrated that SINV replicase can utilize SG promoters from other alphaviruses with variable efficiencies [28]. There is clear evidence that nsP4, the RNA polymerase subunit of the alphavirus replicase, interacts with sequences required for genomic and SG RNA synthesis using different amino acid motifs and depends on the presence of other nsPs [29,30].

Although predicted decades ago [26,31,32] it has only recently been experimentally confirmed that the 5' end of the alphavirus genome contains numerous functional stem-loop (SL) structures. Seven SL structures were identified in the first 300nt of the CHIKV genome. It was shown that some of these structures are important for genome replication in general while others were only important for replication of the virus genome in either mammalian or in mosquito cells [33]. Structural elements located at 5' end region of the alphavirus genome are also important to overcome certain host innate immune responses. For pathogenic alphaviruses they have been shown to alter binding and functioning of IFIT1, a factor responsible for inhibition of translation from RNAs with a 5' cap lacking 2'-O methylation [34]. It has also been revealed that there is a functional connection between replicase proteins and RNA sequences at the ends of the alphavirus genome. In support of this, mutations in replicase proteins can be compensated for by compensatory changes in sequences at the ends of the virus genome [35]. Conversely, mutations in the 3' UTR result in adaptive changes in virus-encoded proteins [23].

The alphavirus replicase possesses a high activity *in trans* and is capable of replicating RNAs containing suitable structures at their 5' and 3' ends including defective interfering (DI) RNAs [36–39]. If the DI RNA contains SG promoter sequences, SG RNAs are also synthesized. These properties have been utilized to develop a packaging systems for alphavirus replicon vectors [40,41] and, more recently, *trans*-replicase systems for different alphaviruses [42–44]. It has also allowed uncoupling of the replicase protein synthesis from its mRNA replication. This property was used to analyse template requirements of SFV and SINV replicases [26]. However, an overall picture of the cross-utilisation of template RNAs by replicases of different alphaviruses and their host-cell type dependence has been lacking, hampering analysis of the organization of alphavirus replicase complex and replicase protein/RNA interactions. Information on the potential for cross-utilisation of template RNAs by related viruses is important for understanding the basic properties of (alpha)virus infection such as superinfection exclusion or rescue.

This study used extremely sensitive *trans*-replicase systems, constructed previously for different alphaviruses [44]. The cross-utilisation of the template RNAs by the different replicases was analysed in both human and mosquito cells. This analysis revealed the existence of templates with different promiscuity. In general, the cross-utilisation of RNA templates was found to be similar in human and mosquito cells. Replicases of alphaviruses not belonging to the SFV complex showed high preference for their own RNA templates, with their capacity for using heterologous templates being more limited in mosquito cells compared to human cells. The sequence determinants responsible for the capacity of SINV, RRV and CHIKV replicases to use each other’s templates were shown to locate in the 5' region of the template RNA. For SINV replicase they mapped to the extreme 5' end of the genome and the first SL structure. Mutations introduced into these elements had a severe impact on template RNA replication and on the rescue of recombinant SINV from infectious transcripts. In addition to revealing the broader picture of RNA template cross-utilisation by alphavirus replicases, our study also provides new and highly efficient tools to generate replicating RNAs that can be used for analysis of their structure in cells. This also opens new possibilities for genetic attenuation of alphaviruses and for development and analysis of compounds targeting critical regions of alphavirus RNA genomes.

## Results

**Fig 1:**
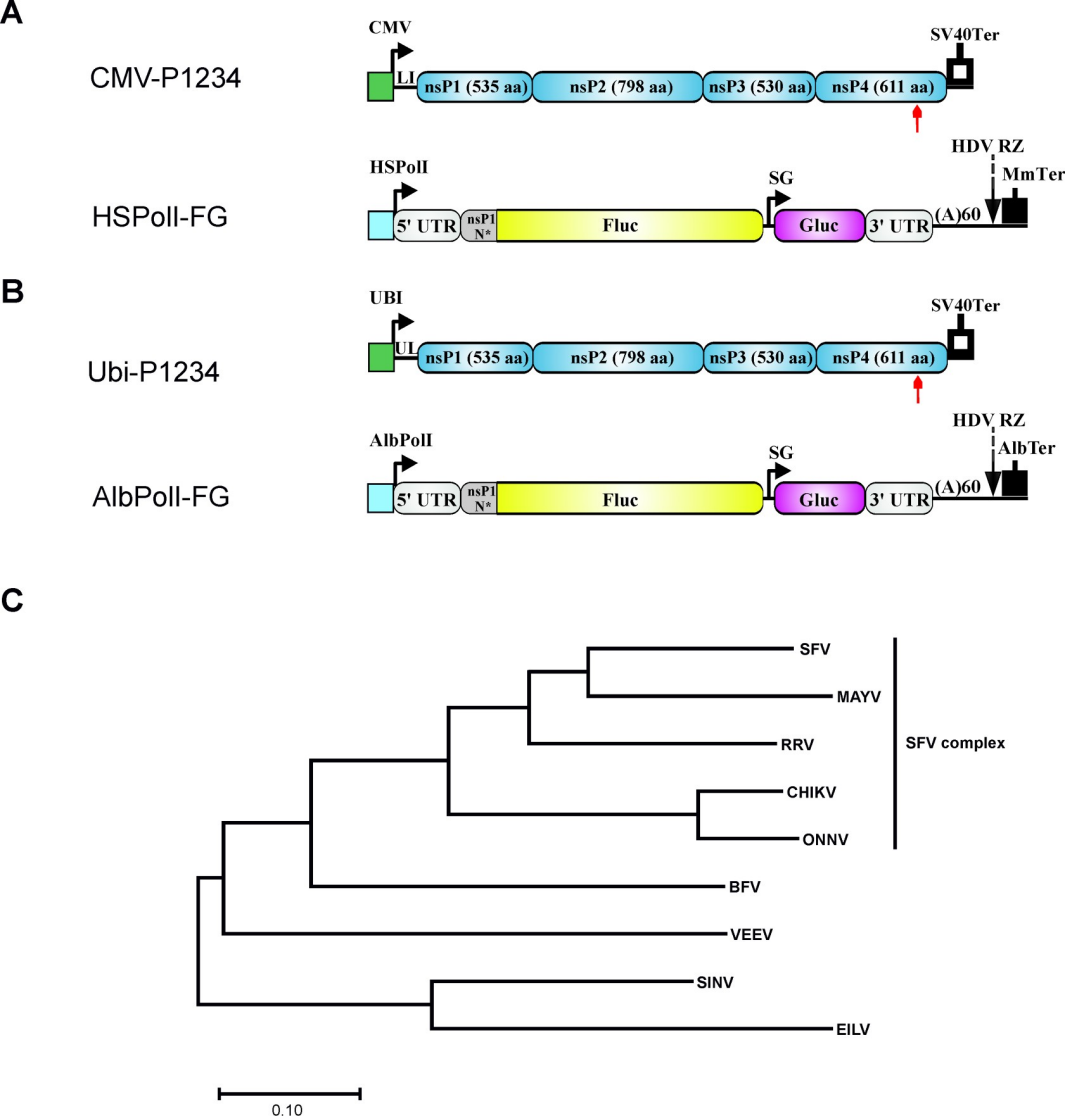
Alphaviruses selected for the analysis and designs of *trans*-replication tools. (A) Schematic representation of constructs for human cells. CMV, immediate early promoter of human cytomegalovirus; LI, leader sequence of the herpes simplex virus thymidine kinase gene with artificial intron; SV40Ter, simian virus 40 late polyadenylation region; HSPolI, a truncated promoter (residues −211 to −1) for human RNA polymerase I; MmTer, a terminator for RNA polymerase I in mice. (B). Schematic representation of constructs for *Aedes albopictus* cells. UBI, polyubiquitin promoter of *Aedes aegypti*; UL, leader sequence of *Aedes aegypti* polyubiquitin gene with a natural intron; AlbPolI–truncated promoter (residues −250 to −1) for *Aedes albopictus* RNA polymerase I; AlbTer–putative terminator for *Aedes albopictus* RNA polymerase I. (A, B) 5′ UTR, full length 5’ UTR of an alphavirus; 3’ UTR, truncated (last 110 residues) 3′ UTR of an alphavirus; SG—SG promoter spanning (with respect to termination codon of nsP4) from position -79 to the end of intergenic region, nsP1 N*—region encoding the N-terminal 77 to 114 amino acid residues of nsP1, depending on the virus; HDV RZ—antisense strand ribozyme of hepatitis delta virus. Red arrow indicates the location of the GDD motif in nsP4; in polymerase negative constructs this was replaced by GAA. The vector backbones are not shown and drawings are not to scale. (C) Phylogenetic tree of replicases of analysed alphaviruses. Phylogenetic tree was constructed using evolutionary analysis by Maximum Likelihood method and JTT matrix based model. The tree is drawn to scale, with branch lengths measured in the number of substitution per site. This analysis involved sequences of P1234 of indicated viruses. Evolutionary analysis was conducted using MEGA-X software.

We have previously shown that replicases of eight arbovirus members of the *Alphavirus* genus are capable of replicating and transcribing their cognate template RNAs in human cells [44]. Here, we additionally created replicase and template RNA expression constructs for the mosquito-specific EILV. In order to allow the analysis of RNA replication in mosquito cells, template RNA and replicase expression plasmids for *Ae albopictus* cells were constructed using designs previously described for CHIKV [45]. Altogether, this resulted in nine sets of template RNA/replicase expression plasmids for each virus in human (Fig 1A) and *Ae albopictus* cells (Fig 1B). In order to compare replication in two different host cell types, replicase expression constructs used in this study were based on native coding sequences rather than host-cell adapted coding sequences.

SFV, MAYV, RRV, CHIKV and ONNV belong to the SFV complex while SINV, VEEV, BFV and EILV represent outgroup alphaviruses. The relative degrees of similarity between the replicases of SFV complex and outgroup viruses are indicated in Fig 1C. The differences between outgroup viruses and those belonging to the SFV complex had an impact on the design of the template RNAs. The 5' region of the CHIKV genome contains seven SL structures that affect genome replication in mammalian and/or mosquito cells [33]. As similar structures can be predicted for RNAs of other members of the SFV complex (S1 Fig), the 5' ends of the template RNAs of SFV, MAYV, RRV and ONNV had an identical design to that of the previously constructed CHIKV template, i.e. the 5' UTR was followed by 231nt encoding the N-terminus of nsP1 [43]. In contrast, the predicted final downstream SL in the 5' genomic region of the outgroup viruses (equivalent to SL246 in CHIKV [33]) align less well or, for BFV and SINV, is predicted to have different secondary structure (S1 Fig). Therefore, based on our previously published predictions for the structure and position of secondary structure elements homologous to those in CHIKV, longer coding regions of nsP1 were incorporated into the template RNAs of these viruses. These had variable lengths, i.e. 258nt for EILV, 267nt for VEEV, 339nt for BFV and 342nt for SINV. For simplicity, hereafter the full-length RNA serving as template for Fluc expression is termed “genomic RNA” (and its synthesis as “replication”), the RNA synthesized from the SG promoter serving as template for Gluc expression is termed “SG RNA” (and its synthesis as “transcription”) and all RNAs synthesized by *trans*-replicases are referred to as “viral RNAs”. The levels of Fluc and Gluc expression in human cells, transfected with plasmids expressing template RNA and corresponding polymerase negative control replicase (P1234^GAA^), were similar for *trans-*replicases derived from different viruses and the same was observed for *Ae albopictus* cells. Therefore, the efficiency of replication and transcription were estimated by fold changes (“boost”) of corresponding reporter expression i.e. reporter activity in cells expressing native P1234 of alphavirus relative to those expressing its polymerase-negative P1234^GAA^ variant.

### *Trans*-replicases of nine alphaviruses are active in human cells

**Fig 2:**
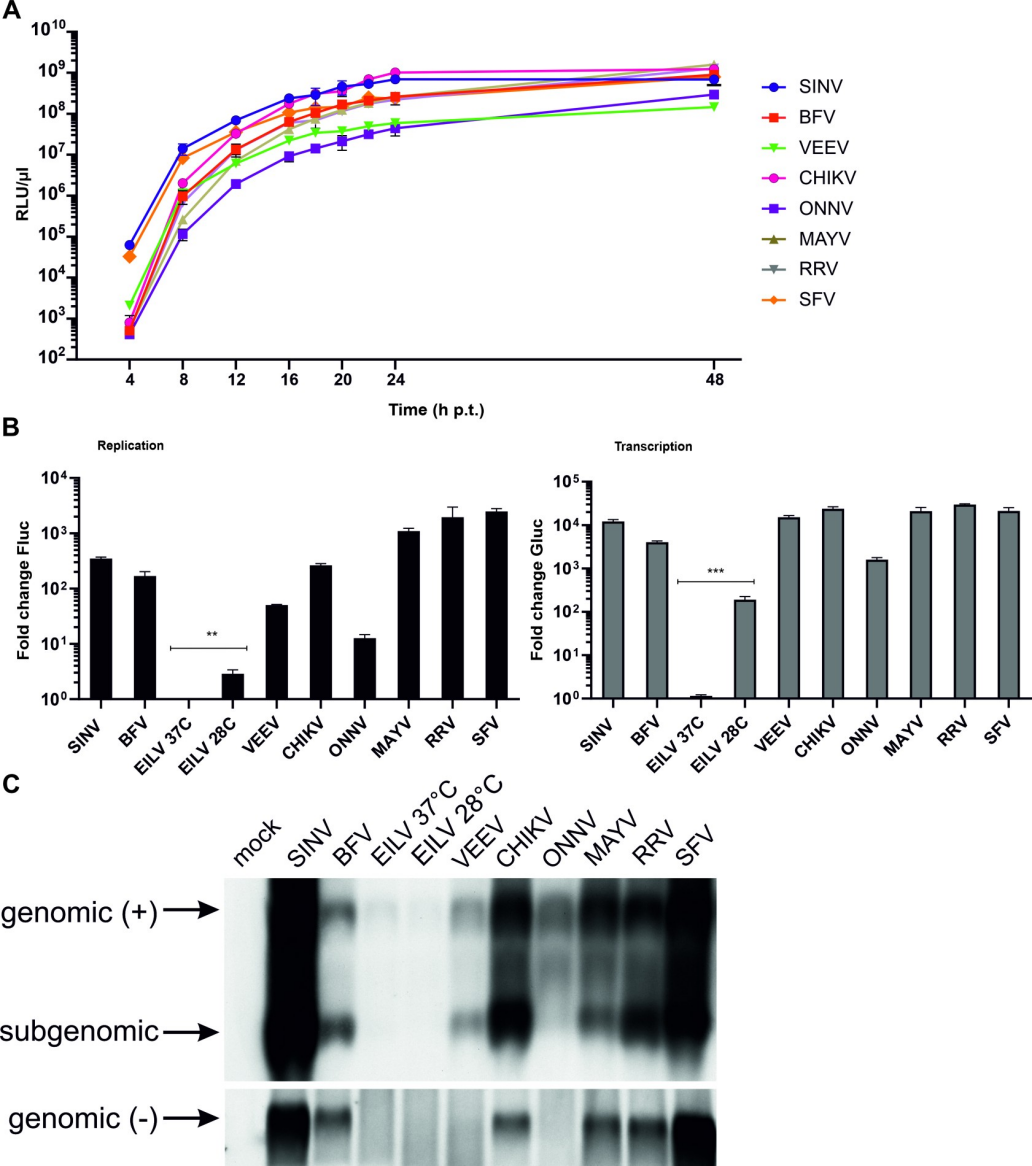
Alphavirus replicases can replicate and transcribe their templates in human cells. (A) HEK293T cells in 12-well plates were co-transfected with matching pairs of CMV-P1234 and HSPolI-FG plasmids. At 4, 8, 12, 16, 20, 22, 24 and 48 h p.t. growth media aliquots were collected the activity of secreted Gluc measured. Means of relative luminescence units (RLU) per 1 μl of sample + standard deviation (SD) of three independent experiments are shown. (B) HEK293T cells in 96-well plates were co-transfected with matching pairs of CMV-P1234 and HSPolI-FG plasmids and, as negative control, CMV-P1234^GAA^, which lacks polymerase activity, instead of CMV-P1234. Cells were incubated at 37°C and lysed 18 h post transfection (p.t.); cells transfected with plasmids containing sequences from EILV were also incubated at 28°C and lysed 48 h p.t. Fluc (marker of replication, left panel) and Gluc (marker of transcription, right panel) activities produced by active replicases were normalized to the P1234^GAA^ controls. Value obtained for P1234^GAA^ controls was taken as 1; activities lower than that observed for P1234^GAA^ are also shown as 1. Means + SD of three independent experiments are shown; p<0.01**; p<0.001*** (Student's unpaired t-test). (C) HEK293T cells in 12-well plates were co-transfected and incubated as described for panel A; control cells were mock-transfected. Total RNA was extracted and analysed by northern blotting. Full-length “genomic” template RNA of positive (+) and negative (-) polarity and subgenomic RNA are indicated. Note that transcripts made by human RNA polymerase I using HSPolI-FG plasmids as templates co-migrate with replicase-generated positive-strand genomic RNA and are detected by the same probe. The experiment was repeated twice with similar results; data from one experiment is shown.

In human cells, the expression kinetics of the Gluc marker were highly similar for all eight *trans*-replicases of arbovirus members of the genus (Fig 2A). Based on these data, a single time point, set at 18 h post transfection (h p.t.), was used in subsequent experiments. As expected, the mosquito-restricted EILV replicase was inactive at 37°C, however, when the experiment was performed at 28°C, significant activity was observed (Fig 2B). Thus, the EILV replicase has a temperature-sensitive phenotype in human cells, but has no absolute requirement for mosquito-specific factors. With two exceptions, the replicases of all analyzed viruses boosted the expression of Fluc and Gluc markers to high levels. The activity of ONNV replicase was clearly lower than that of other replicases, changing the time point for measurement (Fig 2A) or the ratio of replicase and template RNA expression plasmids failed to increase the boost in marker expression. It cannot be excluded that the low efficiency reflects a low level of ONNV replicase expression, for example due to rare codons and/or cryptic splicing sequences present in the coding sequence. However, it is more likely that the relatively low activity of the ONNV *trans*-replicase is a feature caused by the specific properties of ONNV P1234. VEEV *trans*-replicase, albeit efficiently boosting expression of Gluc, was relatively less efficient in boosting expression of Fluc, indicating only modest template RNA replication (Fig 2B).

In order to confirm that the quantification of Fluc and Gluc activities indeed correlate with the synthesis of viral RNAs, RNA synthesis was analyzed using northern blot. The observed levels of viral RNAs clearly correlated with those deduced from analysis of reporter activities. All highly active *trans*-replicases synthesized high levels of negative and positive strand RNAs. In contrast, synthesis of viral RNAs by EILV replicase was below the limit of detection at both 28°C and 37°C. Synthesis of negative strand RNAs by ONNV and VEEV replicases was also close to the detection level. In contrast, positive strands made by these replicases were clearly detected, although ONNV replicase was found to synthesize relatively little SG RNA (Fig 2C), which is consistent with the modest boost of Gluc expression (Fig 2B) as well as with its absolute activity (Fig 2A). The amount of SG RNA made by the VEEV replicase was much lower compared to the amounts of SG RNAs made by replicases of SINV or SFV. Compared with SINV or SFV there was approximately 10-fold difference in Gluc expression (Fig 2A), this is however less prominent than the differences observed between corresponding SG RNA levels (Fig 2C). Furthermore, the boosts of Gluc expression observed for the replicases of VEEV, SINV and SFV were similar (compare Fig 2B and 2C). Most likely the somewhat elevated expression levels of Gluc in the VEEV *trans*-replication system originated from basic properties of the VEEV replicase. nsP2 and nsP3 proteins of Old World alphaviruses inhibit transcription and translation in vertebrate cells [46,47]. In New World alphaviruses the most prominent inducer of cytotoxic effects is the capsid protein [8] which is absent in the VEEV *trans*-replicase. It has been observed that in the absence of host cell shutdown the SG RNAs of alphaviruses lose their competitive advantage over the host cell mRNAs but, nonetheless, the absolute translation efficiency of the SG RNA increases [48–50]. Therefore, it is reasonable to assume that SG RNAs made by the VEEV replicase are more efficiently translated than those made by *trans*-replicases of Old World alphaviruses.

### Alphavirus *trans*-replicase activity in *Aedes albopictus* cells

**Fig 3:**
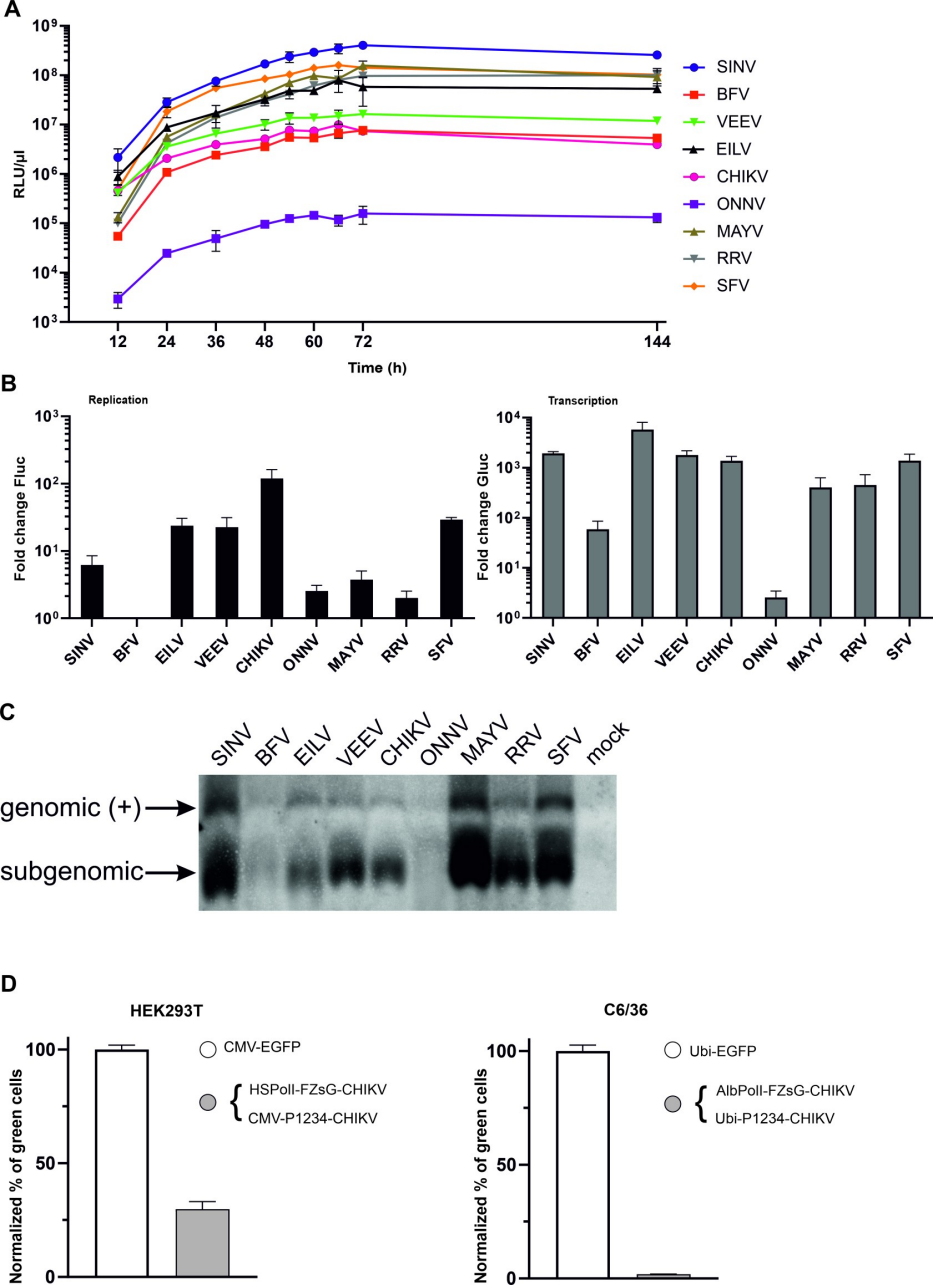
Alphavirus replicases can replicate and transcribe their templates in *Aedes albopictus* cells. (A) C6/36 cells in 12-well plates were co-transfected with matching pairs of Ubi-P1234 and AlbPolI-FG plasmids. At 12, 24. 36, 48, 54, 66, 72 and 144 h p.t. growth media aliquots were collected the activity of secreted Gluc measured. Means of relative luminescence units (RLU) per 1 μl of sample + SD of three independent experiments are shown. (B) C6/36 cells in 96-well plates were co-transfected with matching pairs of AlbPolI-FG and either Ubi-P1234 or Ubi-P1234^GAA^ plasmids. Cells were incubated at 28°C and lysed 48 h p.t. The Fluc (left panel) and Gluc (right panel) activities produced by P1234 replicases were normalized to the polymerase-defective P1234^GAA^ controls as described for Fig 2. Means + SD of three independent experiments are shown. (C) C6/36 cells in 12-well plates were co-transfected as described for panel A. Total RNA was extracted and positive strand RNAs were detected using northern blotting. “Genomic” indicates the full-length template RNA. Note that transcripts made by mosquito RNA polymerase I using AlbPolI-FG plasmids as template co-migrate with replicase-generated positive-strand genomic RNAs. “Subgenomic” indicates the SG RNAs synthesized by replicases using the SG promoter. The experiment was repeated twice with similar results; data from one experiment is shown. (D) HEK293T cells (left panel) were co-transfected with HSPolI-FZsG-CHIKV and CMV-P1234-CHIKV; C6/36 cells (right panel) were co-transfected with AlbPolI-FZsG-CHIKV and Ubi-P1234-CHIKV. Control cells were transfected with plasmid expressing EGFP from a CMV promoter (HEK293T) or from a polyubiquitin promoter (C6/36). At 18 h (HEK293T) or 48 h (C6/36) p.t. cells were collected and analyzed with an Attune NxT Acoustic Focusing Cytometer. The number of ZsGreen-expressing cells is shown as a proportion of the number of EGFP expressing cells, to control for the different transfection efficiency of the two cell types. All transfections were performed in triplicate, means + SD are shown.

Except for ONNV all *trans*-replicases were found to display similar Gluc expression profiles in *Ae albopictus* cells (Fig 3A), enabling us to use a single time point, set at 48 h p.t., for subsequent experiments performed in C6/36 cells. Analysis performed in C6/36 cells also revealed that the activities of alphavirus *trans*-replicases were approximately 10-40-fold lower than in human cells. The boost of Fluc expression was typically as low as 10-fold while the boost of Gluc expression was around 1000-fold. Not surprisingly, the exception to this rule was the EILV *trans-*replicase, for which both replication and transcription in C6/36 cells were higher than these obtained in human cells under similar conditions (compare Figs 2B and 3B). It was also observed that the *trans*-replicase activities differed across different alphaviruses. Notably, the activation of Gluc and Fluc expression by ONNV replicase occurred only at very low level (Fig 3B). Combined with low absolute levels of Gluc expression (Fig 3A) this indicates that ONNV *trans-*replicase was virtually inactive in *Ae albopictus* cells. This cell-type specific defect most likely reflects the fact that, in its natural cycle, ONNV is transmitted by *Anopheles* and not by *Aedes* mosquitoes [51]. No boost in Fluc expression and only a very modest boost of Gluc expression were observed with the BFV *trans*-replicase (Fig 3B). Again, low activity may reflect the fact that BFV is mostly transmitted by *Culex annulirostris* or *Aedes vigilax* mosquitoes [52,53]. Interestingly, a very low boost of Fluc expression was also observed for replicases of RRV and MAYV (Fig 3B), which are associated with *Culex annulirostris* and *Aedes vigilax* [54] or *Haemagogus* mosquitos [55], respectively. Thus, there appears to be at least some correlation between vector preference of an alphavirus and the activity of the corresponding *trans*-replicase in C6/36 cells. However, this correlation should not be over-emphasized as for RRV and MAYV the boost of Gluc expression (transcription) in C6/36 cells were relatively high (Fig 3B). Furthermore, alphaviruses can use different vectors for transmission [56] and these viruses are often capable of replicating in cultivated cells of *Aedes* mosquitoes even when the cells are not from a field-relevant vector species.

Consistent with the modest boost of Gluc expression, only very low levels of SG RNA were detected in cells transfected with the BFV *trans*-replicase (Fig 3C). *Trans*-replicases of SFV, CHIKV, RRV, MAYV, VEEV, SINV and EILV synthesized SG RNAs at high levels (Fig 3C). The highest SG RNA levels were observed for SINV, MAYV and SFV *trans*-replicases (Fig 3C), correspondingly the absolute levels of Gluc expression were also highest for these three *trans-*replicases (Fig 3A). Thus, expression of Gluc serves as a reliable marker for replicase-mediated transcription also in *Ae albopictus* cells. Interestingly, however, production of viral genomic RNA was the highest for the SINV and MAYV *trans*-replicases and reasonably high for the RRV replicase despite their relative low boosts of Fluc expression (compare Fig 3B and 3C). Hence, for some of the alphaviruses the boost of Fluc expression in *Ae albopictus* cells cannot be used as a reliable marker for levels of genomic RNAs generated by their *trans*-replicases (compare left panel of Fig 3B and Fig 3C). Consistent with our previous observations [45], levels of negative strand RNAs from transfected C6/36 cells were below the detection limit of northern blot analysis. This analysis also failed to detect any positive-strand viral RNA in cells transfected with the ONNV *trans*-replicase (Fig 3C) confirming that it is, at best, only weakly active in C6/36 cells.

The reduced boosts of reporter expression, the low levels of negative strand RNAs and the poor correlation of the boost in Fluc expression and the genomic RNA synthesis revealed by northern blot all indicate that *trans*-replicases of arboviruses are much less efficient in C6/36 cells than in human cells. When HEK293T and C6/36 cells were transfected with CMV-EGFP and Ubi-EGFP plasmids, respectively, a higher percentage of EGFP positive cells was obtained for HEK293T cells (approximately 43%) than for C6/36 cells (approximately 21%). Next, the Gluc marker in the HSPolI-FG-CHIKV and AlbPolI-FG-CHIKV plasmids was replaced with a ZsGreen marker. Experiments performed with the CHIKV *trans-*replicase revealed that approximately 12% of human but only 0.4% of mosquito cells become ZsGreen positive. At the same time the expression levels of ZsGreen in replication-positive human and mosquito cells were similar (S2 Fig, S3 Fig). Taken together, these data indicate that lower activities of *trans*-replicases in *Ae albopictus* cells were due to a reduced number of cells in which RNA replication was initiated, rather than low levels of replication per cell. Normalizing for transfection efficiency, it was estimated that the template RNA replication was initiated in ~28% of transfected HEK293T cells but only in ~1.9% of transfected C6/36 cells (Fig 3D).

### Replicases are capable of cross-utilizing template RNAs in human cells

The *trans*-replicase systems allow i) analysis of RNA replication activities for the various alphaviruses, independent of entry-related host cell restrictions and factors associated with translation, as well as ii) analyses in the absence of virus adaptation. These properties permitted the comparative analysis of templates originating from the same alphavirus (hereafter “homologous template” or “homologous combination”) to those of other alphaviruses (hereafter “heterologous templates” or “heterologous combinations”). This allowed, for the first time, an extensive analysis of cross-utilization of RNA templates by alphavirus replicases.

In order to include the most distant known member of the genus *Alphavirus*, the fish-infecting salmonid alphavirus (SAV), a HSPolI-FG-SAV template was additionally constructed and used together with the nine templates described in the previous sections. However, it was found that none of the heterologous replicases was able to use the SAV template RNA. To characterize the reasons behind this effect, a working functional homologous combination of SAV replicase and template would be needed but as we were lacking the facilities to cultivate fish cells this could not be obtained. Therefore, the exact reasons why replicases of other alphaviruses lack the capacity to use SAV template RNA remain unknown. It can be speculated that these reasons may include use of unsuitable host cells (human instead of fish cells), differences in critical *cis-*sequences required for SAV replication, temperature (SAV is normally propagated at temperatures below 16°C) affecting formation of essential RNA secondary structures, and/or very high specificity of the SAV template to its own replicase.

**Fig 4:**
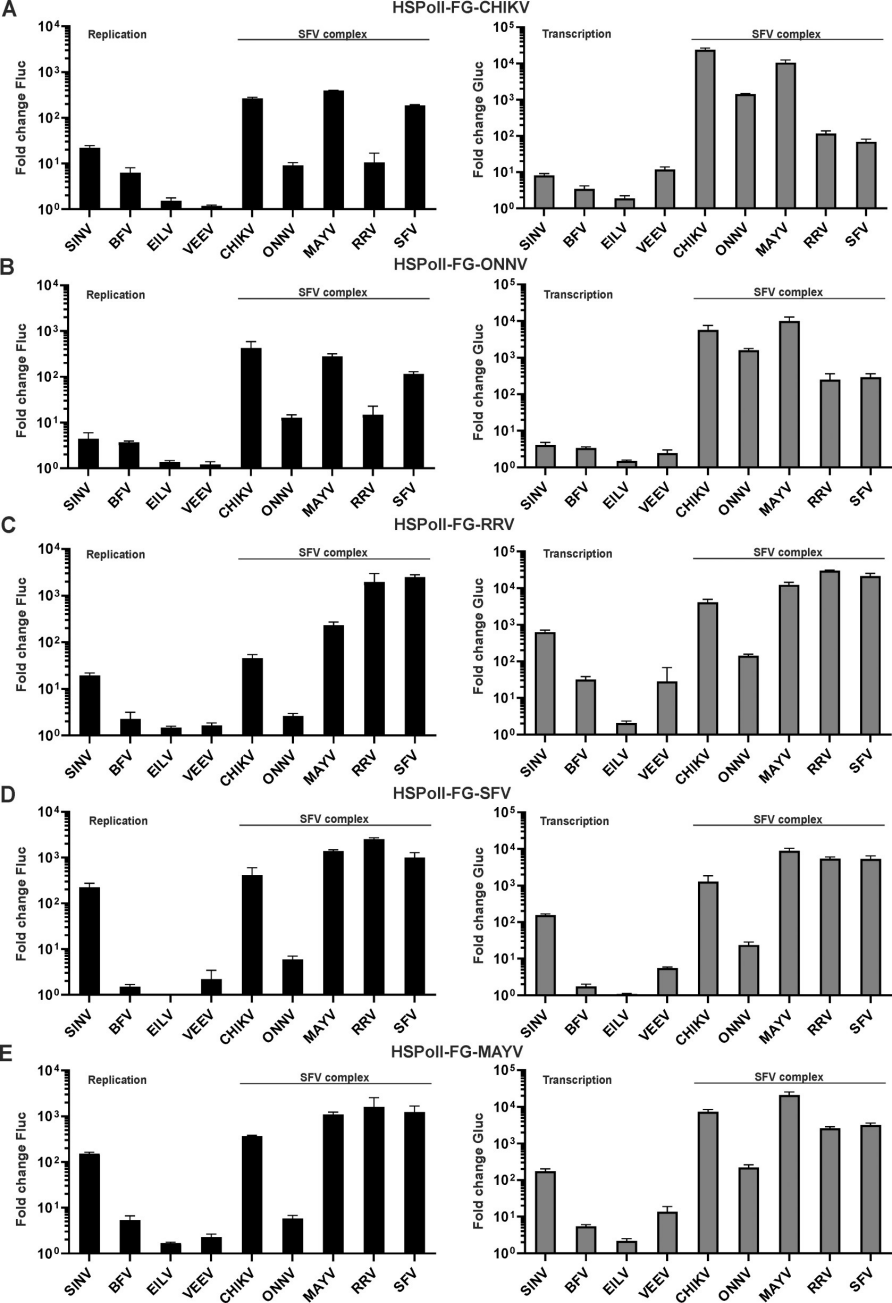
Cross-utilization of templates of viruses belonging to the SFV complex in human cells. HEK293T cells were co-transfected with combinations of each CMV-P1234 replicase expression plasmid or corresponding CMV-P1234^GAA^ control and (A) HSPolI-FG-CHIKV, (B) HSPolI-FG-ONNV, (C) HSPolI-FG-RRV, (D) HSPolI-FG-SFV or (E) HSPolI-FG-MAYV. Transfected cells were incubated at 37°C and lysed 18 h p.t.; cells transfected with plasmids originating from EILV were incubated at 28°C and lysed 48 h p.t. Data represent the luciferase activity (Fluc and Gluc) from CMV-P1234 transfected cells normalized to the paired CMV-P1234^GAA^ control cells. Value obtained for P1234^GAA^ controls was taken as 1; activities lower than that observed for P1234^GAA^ are also shown as 1. X-axis represents different replicases, means + SD are from three independent experiments.

In contrast to the SAV template, templates from other alphaviruses could be cross-utilized by heterologous replicases. In general, replicases of viruses belonging to the SFV complex had the capacity to use each other’s templates (Fig 4A–4E; S4 Fig). A correlation with the phylogenetic relationship of these viruses was also observed within the group. Thus, replicases of CHIKV and ONNV used each other’s templates efficiently; furthermore, the use of CHIKV and ONNV templates by replicases of other alphaviruses was very similar (compare Fig 4A and 4B). These templates were also efficiently used by MAYV replicase and, to a lesser extent, by replicases of RRV and SFV. On HSPolI-FG-CHIKV the transcription efficiencies of RRV and SFV replicases were significantly lower than CHIKV replicase (p<0.0001 for both of SFV and RRV). The same was also observed for HSPolI-FG-ONNV template which was transcribed significantly more efficient by ONNV replicase then by SFV or RRV replicases (p<0.001 at both cases). In addition, compared to CHIKV replicase, the RRV replicase was significantly less efficient for replication of CHIKV and ONNV template RNAs (Fig 4A and 4B) (p<0.0001 and p<0.001 respectively). Template RNAs of RRV and SFV also behaved similar to each other (compare Fig 4C and 4D) and, with the exception of ONNV, were efficiently transcribed by replicases of viruses from the SFV complex. Replication of these template RNAs was also similar, except that CHIKV and MAYV replicases used the SFV template significantly more efficient (p<0.05 for CHIKV and p<0.001 for MAYV replicase) than the RRV template (compare Fig 4C and 4D). Again, with the exception of that of ONNV, the MAYV template was efficiently replicated by replicases from viruses belonging to the SFV complex. For transcription it was also noted that RRV and SFV replicases used this template significantly less efficient than the CHIKV replicase (p<0.01 at both cases), indicating that the MAYV SG promoter is not optimal for SFV and RRV replicases (Fig 4E). Taken together, it can be concluded that viruses belonging to the SFV complex can replicate and transcribe each other’s templates. In contrast, no replicase of the outgroup viruses was capable of comparable utilization of any of the SFV-complex templates. The highest replication efficiencies were observed for the SINV replicase on MAYV and SFV templates (S5 Fig). Utilization of the CHIKV and especially ONNV template by replicases of outgroup viruses was very inefficient (Fig 4A–4D). Cross-utilization of SFV and SINV templates has been previously studied using corresponding replicon vectors and DI RNA type reporters. In contrast to our findings (Fig 4D) this analysis revealed that SINV replicase was virtually unable to use a SFV template [26]. The differences probably originate from the use of different tools, including the use of self-replicating RNA as source of replicase, in the earlier experiments.

**Fig 5:**
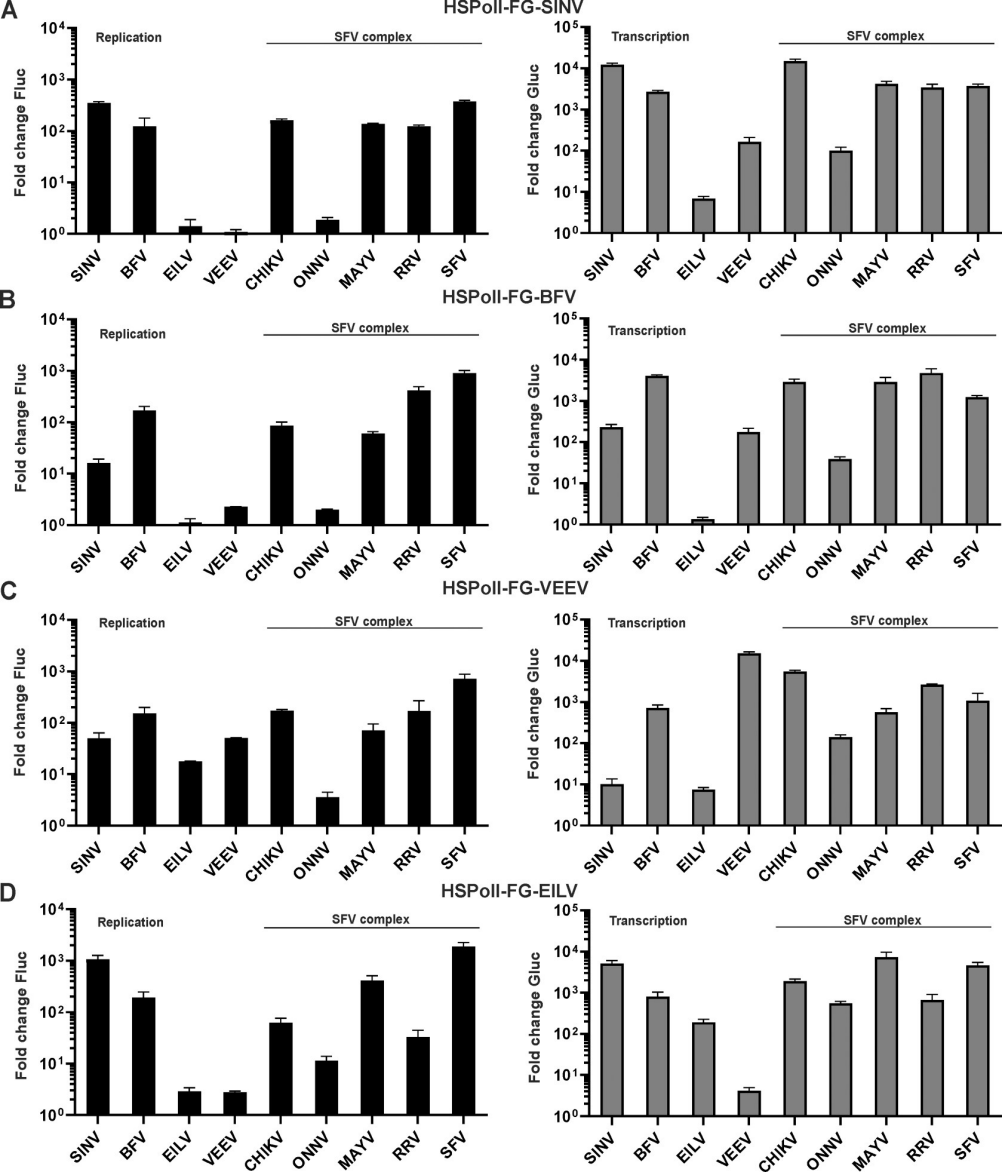
Cross-utilization of templates of outgroup alphaviruses in human cells. HEK293T cells were co-transfected with combinations of each CMV-P1234 replicase expression plasmid or corresponding CMV-P1234^GAA^ control and (A) HSPolI-FG-SINV, (B) HSPolI-FG-BFV, (C) HSPolI-FG-VEEV or (D) HSPolI-FG-EILV. Transfected cells were incubated at 37°C and lysed 18 h p.t.; cells transfected with plasmids originating from EILV were incubated at 28°C and lysed 48 h p.t. Data represent the luciferase activity (Fluc and Gluc) from CMV-P1234 transfected cells normalized to the paired CMV-P1234^GAA^ control cells. Value obtained for P1234^GAA^ controls was taken as 1; activities lower than that observed for P1234^GAA^ are also shown as 1. X-axis represents different replicases, means + SD are from three independent experiments.

The utilization of RNA templates of outgroup viruses was clearly different. Surprisingly, the templates of these evolutionary distant alphaviruses (Fig 1C) behaved rather similar to each other. The SINV and BFV templates were efficiently used by replicases of all viruses except EILV, VEEV and ONNV (Fig 5A and 5B). The VEEV template was replicated well by all replicases except that of ONNV and at least three of the heterologous replicases (BFV, CHIKV, and SFV) significantly outperformed the homologous one (p<0.05 for BFV, p<0.01 for CHIKV and SFV replicases). The boost of Gluc expression was, however, greatest for the homologous replicase (p<0.001 compared to any heterologous replicase), an effect that may be the result of the lower cytotoxicity of VEEV non-structural proteins for human cells. Only the SINV and EILV replicases failed to transcribe the VEEV template (Fig 5C), likely because of the inability to use the VEEV SG promoter. Taken together, it could be concluded that in contrast to templates from viruses belonging to the SFV complex the templates of SINV, BFV and VEEV can be efficiently used, albeit with some exceptions, by replicases of different alphaviruses. Surprisingly, it was found that the same also applies to the template RNA of insect specific EILV. This template was efficiently used by replicases from all alphaviruses except VEEV (Fig 5D). Replication of the template by EILV and VEEV replicases was inefficient and not significantly different between the two (p>0.05). In contrast, transcription of the template was clearly and significantly (p<0.001) more efficient for homologous replicase (Fig 5D). Overall, the use of EILV template was very similar to that of SINV (compare Fig 5A and 5D), supporting the observation that these viruses are phylogenetically related (Fig 1C) and harbor similar secondary structures at the 5’ end region of genome (S1 Fig).

**Fig 6:**
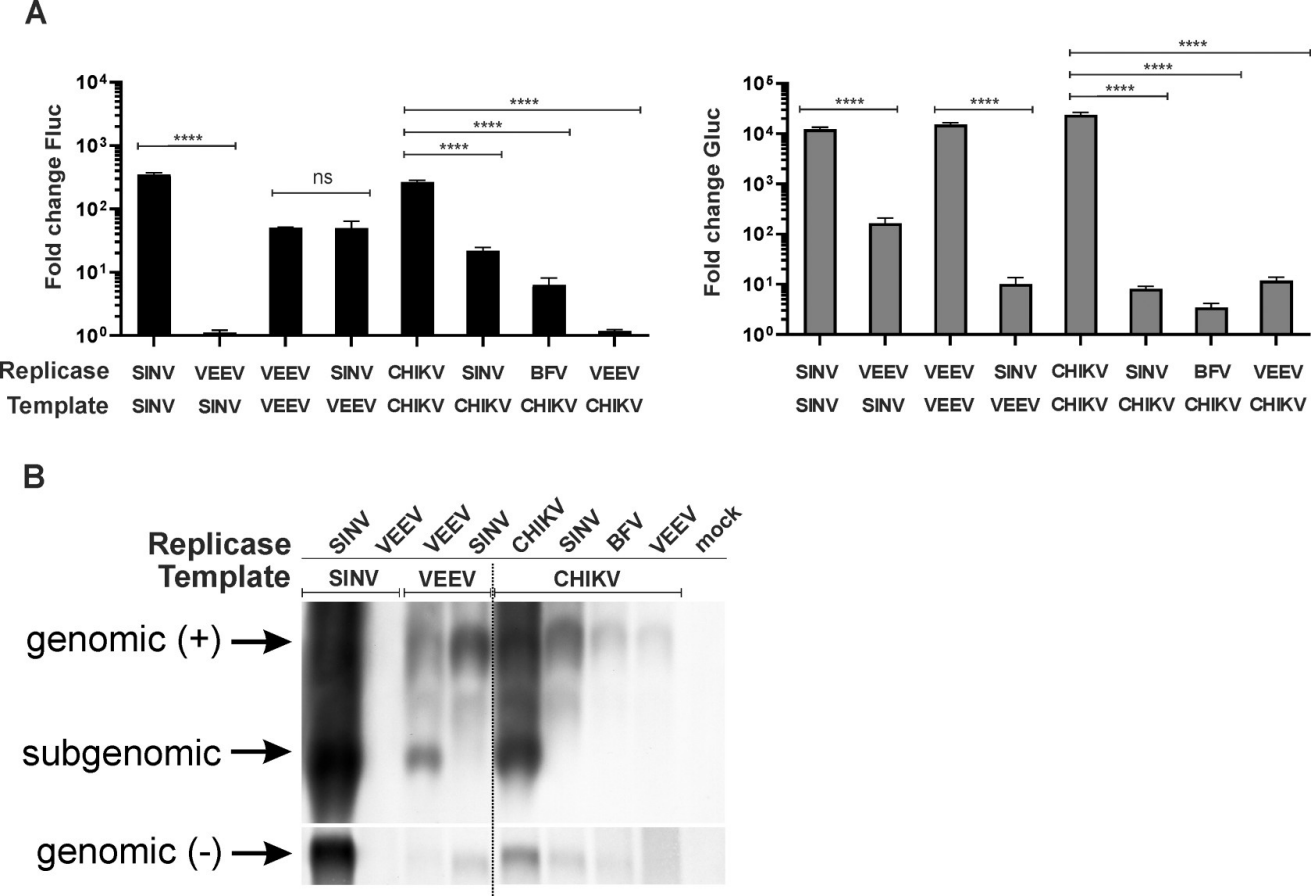
Incompatible combinations of alphavirus replicases and templates have defects in synthesis of viral RNAs. (A) HEK293T cells were co-transfected with combinations of HSPolI-FG template and CMV-P1234 replicase expression plasmids as indicated; in control cells CMV-P1234 was replaced with CMV-P1234^GAA^. Cells were lysed 18 h p.t. Data represent the luciferase activity (Fluc and Gluc) from CMV-P1234 transfected cells normalized to the paired CMV-P1234^GAA^ control cells. Data is replotted from Fig 4 and Fig 5. Means + SD are from three independent experiments; ns, not significant, ****p<0.0001 (Student's unpaired t-test). (B) HEK293T cells were co-transfected with combinations of CMV-P1234 and HSPolI-FG plasmids as indicated. At 18 h p.t. cells were lysed and total RNAs were isolated. RNAs were analysed by northern blot as described for Fig 2B. The experiment was repeated twice with similar results; data from one experiment is shown.

In order to reveal the molecular basis of incompatibility of some replicase/template pairs an analysis of viral RNAs was performed. It was concluded from the reporter expression that the VEEV replicase cannot use the SINV template while the SINV replicase can only replicate, but not transcribe, the VEEV template (Fig 6A; S5 Fig). These effects were confirmed by analysis of the viral RNAs. The VEEV replicase was found to be unable to synthesize any detectable RNA using the SINV template while the SINV replicase efficiently synthesized VEEV negative and positive strand genomic RNAs but failed to synthesize SG RNA (Fig 6B). Similarly, analysis of reporter expression lead to the conclusion that the CHIKV template can be replicated by the SINV or BFV but not by the VEEV replicases and that none of them was able to use the CHIKV SG promoter (Fig 6A). Again, the data obtained using northern blot fully supported these conclusions (compare Fig 6A and 6B). In contrast to VEEV, the replicases of SINV, and to lesser extent of BFV, were indeed able to synthesize negative and positive genomic RNAs from the CHIKV template. At the same time, none of these heterologous replicases produced CHIKV SG RNA at detectable levels. Thus, the SG promoter of many alphaviruses is rather selective: the VEEV replicase cannot use the SG promoter of SINV, and the SINV and BFV replicases cannot use the SG promoter of CHIKV. The effect was not reciprocal as the CHIKV replicase used the SG promoters of SINV and BFV as efficiently as the homologous replicases (Fig 5A and 5B, in both cases p>0.05).

### Alphavirus replicases display lower capacity to use heterologous templates in *Aedes albopictus* cells

**Fig 7:**
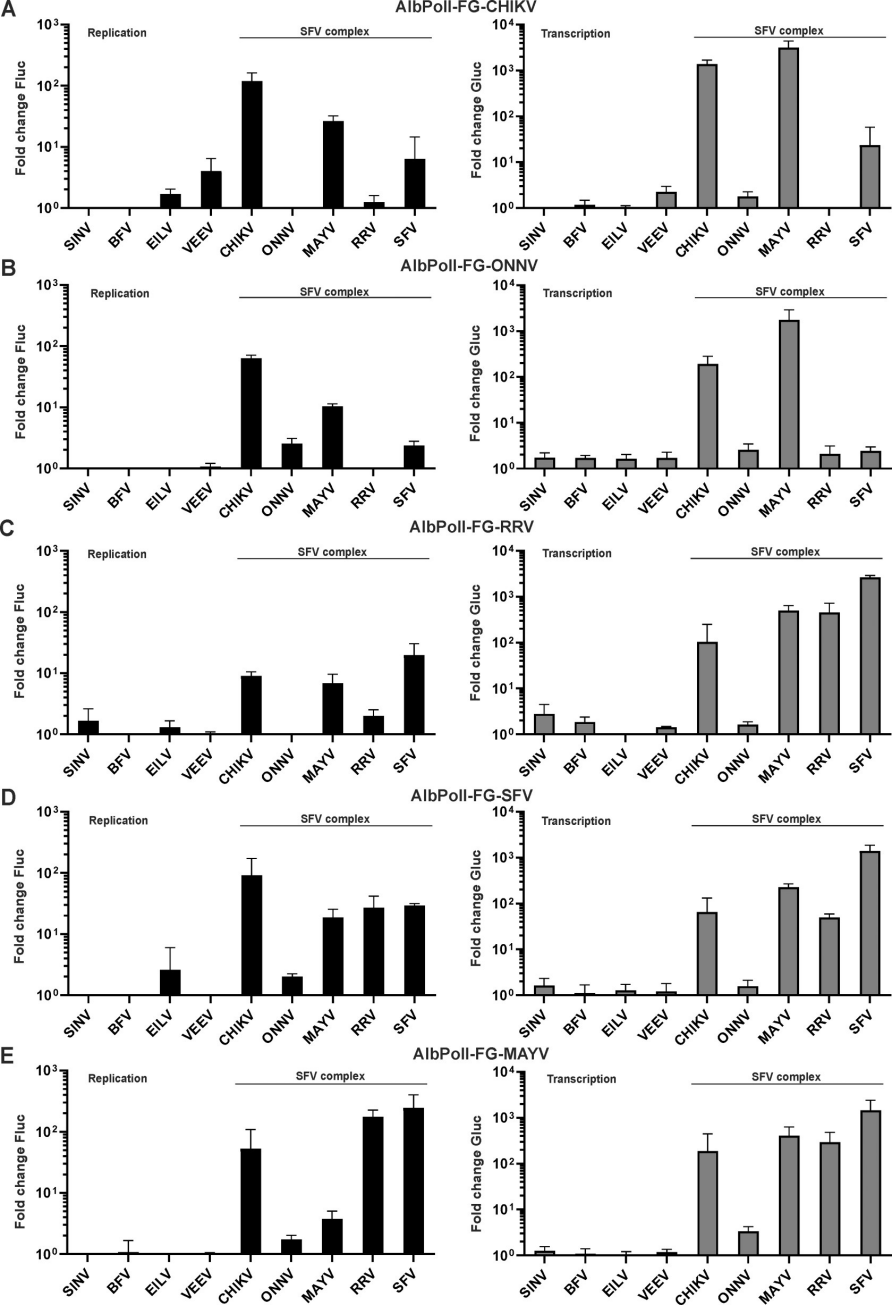
Cross-utilization of templates of viruses belonging to the SFV complex in *Aedes albopictus* C6/36 cells. C6/36 cells were co-transfected with combinations of each Ubi-P1234 replicase expression plasmid or corresponding Ubi-P1234^GAA^ control and (A) AlbPolI-FG-CHIKV, (B) AlbPolI-FG-ONNV, (C) AlbPolI-FG-RRV, (D) AlbPolI-FG-SFV or (E) AlbPolI-FG-MAYV. Transfected cells were incubated at 28°C and lysed 48 h p.t. Data represent the luciferase activity (Fluc and Gluc) from Ubi-P1234 transfected cells normalized to the paired Ubi-P1234^GAA^ control cells. Value obtained for P1234^GAA^ controls was taken as 1; activities lower than that observed for P1234^GAA^ are also shown as 1. X-axis represents different replicases, means + SD from three independent experiments are shown.

**Fig 8:**
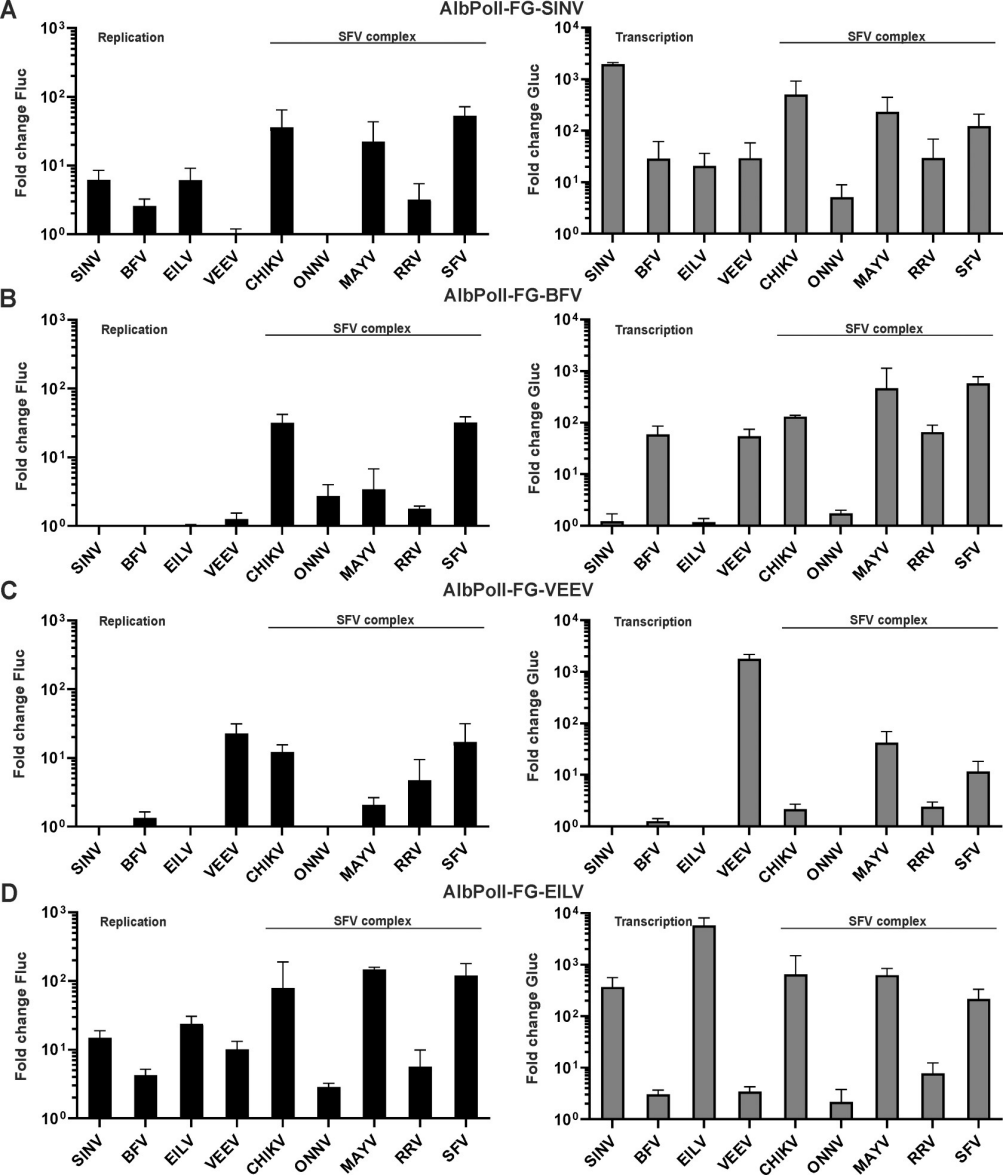
Cross-utilization of templates of outgroup alphaviruses in *Aedes albopictus* C6/36 cells. C6/36 cells were co-transfected with combinations of each Ubi-P1234 replicase expression plasmid or corresponding Ubi-P1234^GAA^ control and (A) AlbPolI-FG-SINV, (B) AlbPolI-FG-BFV, (C) AlbPolI-FG-VEEV or (D) AlbPolI-FG-EILV. Transfected cells were incubated at 28°C and lysed 48 h p.t. Data represent the luciferase activity (Fluc and Gluc) from Ubi-P1234 transfected cells normalized to the paired Ubi-P1234^GAA^ control cells. Value obtained for P1234^GAA^ controls was taken as 1; activities lower than that observed for P1234^GAA^ are also shown as 1. X-axis represents different replicases, means + SD from three independent experiments are shown.

Previous studies have shown that *cis*-active sequences and/or RNA secondary structures in alphavirus RNAs may have different functionalities in vertebrate and mosquito cells [23,33,57]. Therefore, the cross-utilization experiments described in the section above were also performed in *Ae albopictus* cells. These experiments confirmed that the ONNV replicase was essentially inactive in C6/36 cells (Figs 7 and 8) because the boost of Fluc expression was typically undetectable and the boost of Gluc expression did not exceed 2-3-fold. This finding contrasts sharply with the observation in human cells where the ONNV replicase, albeit typically less efficient than replicases of other viruses, was able to boost Fluc expression approximately 10-fold and Gluc expression >1000-fold (Fig 4A and 4B). The difference in reporter gene expression observed between human and mosquito cells appears too large to be solely attributed to the less efficient initiation of replication in C6/36 cells (Fig 3D) and therefore presumably originate from some intrinsic property of ONNV. At the same time, replicases of CHIKV and MAYV used the ONNV template efficiently (Fig 7B). Thus, the replication defect of ONNV in C6/36 cells was due to the virus replicase and not the template RNA.

Cross-utilization of template RNAs of viruses belonging to the SFV complex in C6/36 cells was similar to that in human cells except that differences between alternative replicases were more pronounced, possibly due to a lower efficiency of *trans*-replicases in C6/36 cells. Templates of CHIKV and ONNV were efficiently replicated and transcribed by CHIKV and MAYV replicases. CHIKV template was used, albeit less efficient, also by the replicase of SFV. The RRV replicase was virtually unable to use either of these templates (Fig 7A and 7B). In contrast, the templates of SFV, RRV and MAYV were similarly and efficiently replicated and transcribed by all viral replicases (except the one from ONNV) of the SFV complex (Fig 7C–7E). For the replicases of RRV and MAYV it was observed that the boost of Fluc expression from the homologous templates was relatively modest and replication of heterologous templates was often much more efficient (S6 Fig). However, the significance of this, if any, is not clear as the effect probably originates from the low sensitivity of the Fluc reporter-based replication assay in C6/36 cells (Fig 3B). No replicase from outgroup alphaviruses was capable of using template RNAs from the SFV complex (Fig 7A–7E).

In C6/36 cells, the SINV template was used in the same way as in human cells. It was efficiently replicated by several heterologous replicases, among which the replicases of CHIKV, MAYV and SFV significantly outperformed the homologous replicase (p<0.05, p<0.05 and p<0.01, respectively). These three replicases were also efficient in SINV template transcription though for this their activities were significantly lower than that of homologous replicase (p<0.01 in all three cases). In contrast to human cells, where the SINV template was also efficiently replicated and transcribed by the RRV replicase, this replicase performed poorly in C6/36 cells (Fig 8A). The template of BFV was in general less efficiently used than other templates. It was replicated relatively efficiently only by replicases of CHIKV and SFV. However, replicases of viruses from the SFV complex (except ONNV), the homologous replicase and that of VEEV transcribed the BFV template (Fig 8B). The VEEV template was efficiently used only by its own replicase and, to a smaller extent, by replicases of viruses from the SFV complex (Fig 8C). Although a similar trend was observed in human cells (Fig 5C), it was evident that in C6/36 cells the VEEV template is preferentially transcribed by the homologous replicase.

The EILV template RNA was efficiently used by SINV, CHIKV, MAYV and SFV replicase and poorly by the VEEV replicase. Unlike the situation in human cells, it was also poorly used by the replicase of BFV, RRV and ONNV (Fig 8D). It was also observed that while the EILV replicase was very active using the homologous template, it failed to use the templates of BFV, VEEV and viruses from SFV complex (S7 Fig). The only heterologous template used by EILV replicase to any extent was that of SINV (Fig 8A, S7 Fig). Thus, in contrast to the promiscuous utilization of EILV template RNA, the EILV replicase has a limited capacity to use heterologous templates.

In summary, our findings revealed similarities and differences of template RNA use in human and *Ae albopictus* cells. In general, in C6/36 cells alphavirus replicases had a reduced capacity to use templates other than their own. This was less pronounced for viruses belonging to the SFV complex but very clear for replicases of outgroup viruses, none of which could use templates of viruses from the SFV complex. It was clear that in C6/36 cells, the replicases of SINV, BFV, VEEV and EILV could typically replicate and transcribe only homologous template RNAs. The only exception was that the SINV replicase could also use the template RNA of EILV (S7 Fig).

### Determinants for specificity of alphavirus template RNA map to its 5' region and SG promoter

In the *trans*-replicase system, replication of the CHIKV template by the SINV replicase was inefficient and no synthesis of SG RNA was observed (Fig 6). At the same time the replicase of CHIKV was capable of using the SINV template efficiently (Fig 5A; S4 Fig). Thus, the cross-utilization of templates by the CHIKV and SINV replicases is, as already described for SFV and SINV [26], not reciprocal. Therefore, the CHIKV and SINV templates were chosen for mapping the determinants important for their use by their corresponding replicases.

To increase the sensitivity of the assay subsequent experiments were performed in U2OS cells that have been found to be the most efficient for CHIKV [43] and SINV *trans*-replicase assays. The 5' regions in the CHIKV and SINV templates used in previous experiments had different lengths, i.e. 307nt for CHIKV and 401nt for SINV. The differences mostly originate from the extra 105nt derived from the nsP1 ORF that were included into the SINV template. Even though this sequence has no known function for alphavirus RNA replication/transcription, we wanted to exclude any possible bias caused by the presence or absence of this region. Therefore, an HSPolI-FG-C*CC plasmid, extending the CHIKV template 5' region to an extent similar to the SINV template, was constructed. It was found that the template RNAs encoded by HSPolI-FG-C*CC and HSPolI-FG-CHIKV were both replicated and transcribed with high efficiency by the CHIKV replicase and with low efficiency by the SINV replicase, and no statistically significant difference between two templates was observed for either replicase.

**Fig 9:**
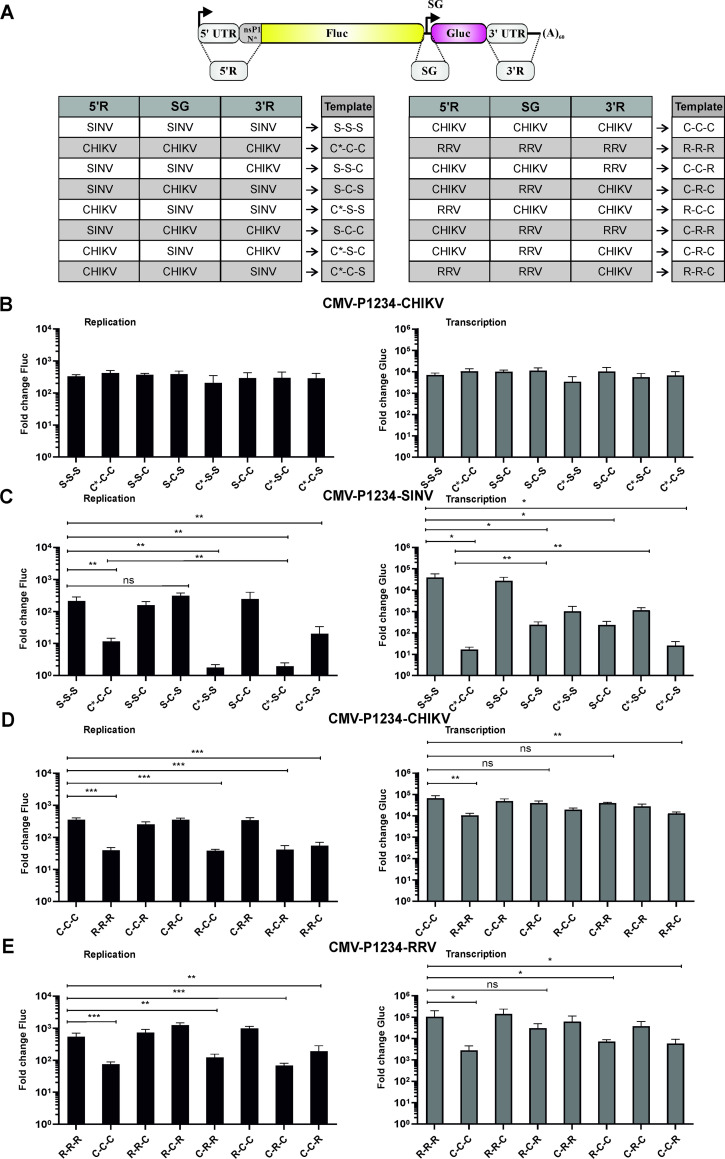
Specificity for the replicase resides in the 5’- and SG regions of the template RNA. (A) Schematic representation and explanation of names of SINV, CHIKV and RRV swapped template RNAs. 5’R—5’ region; 3’R– 3’ region, C* - 5’ region of CHIKV template containing 336nt from nsP1 encoding region; C—5’ region of CHIKV template containing 231nt from nsP1 encoding region. Other elements are the same as on Fig 1A. (B-E) U2OS cells grown in 12-well plate were co-transfected with plasmids encoding for indicated template RNAs and with (B, D) CMV-P1234-CHIKV, (C) CMV-P1234-SINV or (E) CMV-P1234-RRV. For control cells the plasmid expressing active replicase was substituted with corresponding CMV-P1234^GAA^ plasmid. Cells were lysed 18 h p.t. Data represent the luciferase activity (Fluc and Gluc) from CMV-P1234 transfected cells normalized to the paired CMV-P1234^GAA^ control cells. Means + SD are from three (B, C, D) or four (E) independent experiments are shown; ns, not significant, *p<0.05, ** p<0.01, ***p<0.001, ****p<0.0001 (Student's unpaired t-test).

HSPolI-FG-C*CC and HSPolI-FG-SINV were subsequently used for swapping the 5' regions, SG promoters and 3' regions, resulting in six swapped CHIKV/SINV template constructs (Fig 9A). As expected, the CHIKV replicase was able to replicate and transcribe not only the SINV and CHIKV template RNAs but also all six swapped variants (Fig 9B), confirming that it does not discriminate between *cis*-elements in the homologous template and these in the SINV template. At the same time, the SINV replicase replicated and transcribed its own template at high efficiency but was much less efficient on the CHIKV template (Fig 9C). Replacement of the 3' region of SINV with that of the CHIKV template (resulting in S-S-C template) did not reduce the ability of the SINV replicase to use the template (Fig 9C, compare S-S-S and S-S-C). Similarly, swapping of 3' region of the CHIKV template with its counterpart from SINV (resulting in C*-C-S template) did not increase the utilization by the SINV replicase (Fig 9C, compare C*-C-C and C*-C-S). These findings demonstrate that the 3' regions of the CHIKV and SINV templates do not contain any major determinants for SINV replicase specificity. In contrast, swapping of the SINV SG promoter with that of CHIKV drastically diminished the ability of the SINV replicase to transcribe the resulting S-C-S template while replication was slightly but significantly increased (Fig 9C compare S-S-S and S-C-S). The reciprocal swap increased transcription and also significantly reduced the replication of the resulting C*-S-C template by the SINV replicase (Fig 9C, compare C*-C-C and C*-S-C). Taken together these findings confirm that the SINV replicase has a strong preference for its own SG promoter. Furthermore, our data suggests that there may be competition between genomic and SG promoters that leads to reduced replication of the C*-S-C template and somewhat increases replication of the S-C-S template. Consistent with findings using the SFV and SINV templates [26], swapping of their 5' regions had a major effect on replication of corresponding RNAs by the SINV replicase. The C*-S-S template was used very inefficiently while the S-C-C template was replicated by the SINV replicase as efficiently as SINV’s own template RNA (Fig 9C). Thus, the 5' region of the RNA template contains determinants for replication by the SINV replicase.

Next, we wanted to analyze whether these findings also apply for pair of alphaviruses belonging to the SFV complex. Advantage was taken from the finding that both replication and transcription of the CHIKV template by the RRV replicase occurred only at a modest level (Fig 4A). Furthermore, use of the RRV template by the CHIKV replicase was also clearly less efficient than its utilization by the RRV replicase, the difference being especially evident for replication (Fig 4C). It was found that these phenotypes were also preserved in U2OS cells (Fig 9D and 9E). The use of swapped templates again revealed that replacement of the 5' region of the template with its counterpart from a related alphavirus significantly reduced replication of such a template. Replacement of the SG promoter of CHIKV in C-C-C template with that of RRV did not have a significant effect on transcription of such templates. Reciprocal substitutions in the RRV templates somewhat reduced their transcription by the RRV replicase. The observed differences reached statistical significance only in case of the R-C-C and C-C-R templates. The C-C-R template was also characterized by reduced replication, thus its reduced transcription may, at least in part, be due to the swap of the 5' region. The R-C-C template on other hand, replicates similar to the R-R-R template and its reduced transcription is likely due to the presence of a heterologous SG promoter. Finally, swapping of the 3' region of the templates had no detectable effect on replication or transcription of such templates by the CHIKV or RRV replicases (Fig 9D and 9E). Taken together, these experiments confirmed that the determinants responsible for preferential use of templates by replicases of viruses belonging to the SFV complex are also located at the 5' region of the template RNA.

### The beginning of the 5' UTR contains determinants required for efficient replication of RNA templates by SINV replicase

**Fig 10:**
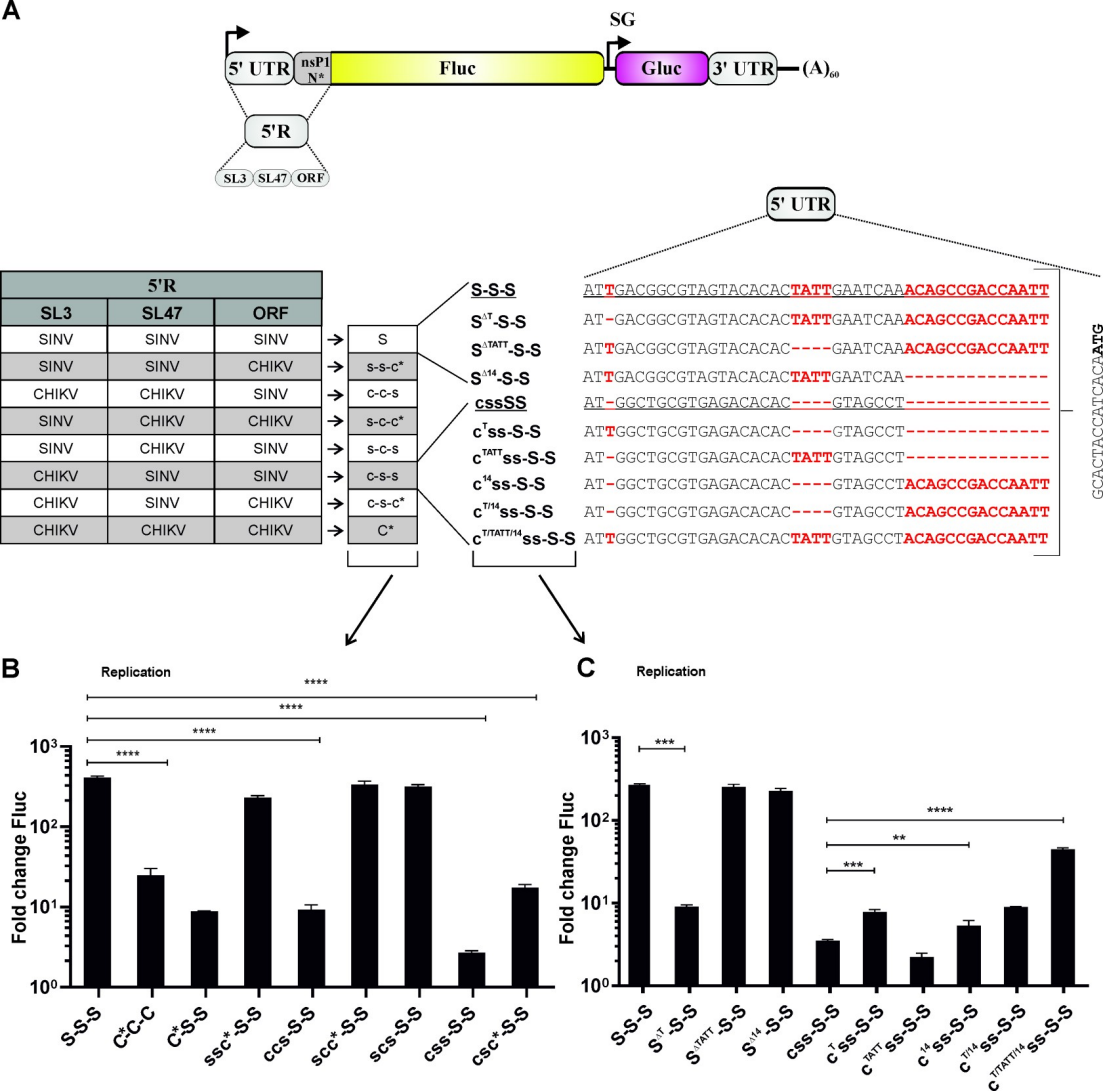
Determinants located at the 5’ end of the template RNA are crucial for SINV replicase. (A). Schematic representation of template RNAs. Changes introduced into the 5’ region of S-S-S template RNA are shown on left panel. Right panel shows 5’ UTR sequences of modified S-S-S and css-S-S templates. SL structures in CHIKV sequence are designated SL3 and SL47 according to [33]. Other elements and designations are the same as Figs 1A and 9A. (B). U2OS cells grown in 12-well plate were co-transfected with CMV-P1234-SINV and HSPolI-FG-SSS, HSPolI-FG-C*CC, HSPolI-FG-C*SS or derivatives of HSPolI-FG-SSS expressing templates containing indicated swaps in their 5' region. For transfection of control cells CMV-P1234^GAA^-SINV and HSPolI-FG-SSS were used. (C) U2OS cells grown on 12-well plate were co-transfected with CMV-P1234-SINV and HSPolI-FG-SSS, HSPolI-FG-cssSS or their derivatives expressing templates containing indicated mutations in the SL3 and/or in extreme 5’ end of the template. For transfection of control cells CMV-P1234^GAA^-SINV was used instead of CMV-P1234-SINV. Cells were lysed 18 h p.t. Fluc activities produced by active replicases were normalized to those measured in control cells. Means + SD of three independent experiments are shown; ** p<0.01, ***p<0.001, ****p<0.0001(Student's unpaired t-test).

Analyses of SINV/SFV chimeric templates have previously convincingly demonstrated that replacement of the SINV 5' UTR with that of SFV drastically reduces the use of the resulting template by the SINV replicase [26]. To analyze whether the same applies for SINV/CHIKV chimeras we took advantage of the SHAPE derived and reverse genetically-verified structure of the 5' end of the CHIKV genome [33] and designed hybrid templates shown in Fig 10A. It was found that templates where the SL3 and SL47 regions (ccs-S-S), the SL3 and the nsP1 ORF regions (csc*-S-S) or only the SL3 region (css-S-S) originated from CHIKV were inefficiently replicated by the SINV replicase, similar to the C*-C-C and C*-S-S templates. In contrast, all chimeric templates that contained the SINV 5' sequences to the end of the SL3 element (ssc*-S-S, scc*-S-S and scs-S-S) were replicated as efficiently as the SINV template by the SINV replicase. Thus, in order to replicate the SINV/CHIKV chimeric templates efficiently, the SINV replicase needs homologous sequences located at the 5' end of the template and/or in SL3. Consistent with earlier findings [32] the sequences of the SINV 5' UTR downstream of nucleotide 45 had little, if any, impact on the replication efficiency.

The comparison of the 5' ends of the efficiently replicated S-S-S template and the very inefficiently replicated css-S-S template highlighted that the former is longer due to three insertions of 1 (T, position 3), 4 (TATT, positions 21–25) and 14 (ACAGCCGACCAATT, positions 32–45) nucleotides (Fig 10A). Therefore, we hypothesized that the shorter length of the 5' UTR may be the reason for the low replication efficiency of the css-S-S template. Indeed, adding the 14 nucleotide fragment to this template resulted in significant increase of its replication efficiency. However, the reciprocal mutation, resulting in the S^Δ14^-S-S template, had no effect on the replication of the truncated SINV template. This contrasts with the previous findings indicating that deletions of nucleotides 36 or 37 results in an impaired, temperature-sensitive, phenotype [32]. The addition of 4 nucleotides to the css-S-S template or their removal from the S-S-S template had minimal, if any, effect on their replication efficiencies. A similar lack of effect has been previously observed for analogous mutations introduced in the SINV genome [32]. In sharp contrast, deletion of a single nucleotide from position 3 of the SINV template drastically reduced the replication efficiency of the resulting RNA (Fig 10C). Deletion of a single nucleotide from position 5 of the SINV template RNA results in a very similar effect [26] and deletion of residues 2–4 of the SINV genome is lethal for the virus [32]. Thus, it should be concluded that the length and/or sequence of the 5' extreme of SINV genome is crucial for the use of the template RNA by the SINV replicase. Interestingly however, experiments using the css-S-S template only partly confirmed this hypothesis. Addition of a T residue after nucleotide 2 in the construct for expression of this template RNA resulted in a minor but significant increase of its replication efficiency. The same was observed when this change was combined with the addition of a 14nt sequence. Only the addition of a third missing element, the TATT sequence, resulted in a prominent increase of the replication efficiency of the corresponding template RNA (Fig 10C). This data indicates that for conversion of the CHIKV-type template to the SINV-type the synergistic effect of several insertions was required. All the effects described above were specific for the SINV replicase as the CHIKV replicase used all templates shown in Fig 10A with high and very similar efficiencies.

The *trans*-replicase assays, though very efficient and sensitive, are based on an artificial system. To exclude the possibility that the observed effects may represent artifacts of the system we introduced the selected mutations into an infectious cDNA plasmid of SINV resulting in plasmids designated pToto1101^ΔT^, pToto1101^css^ and pToto1101^cT/TATT/14ss^. When the effects of the introduced mutations were analysed using an infectious centre assay (ICA) it was found that transcripts from pToto1101^cT/TATT/14ss^ had an infectivity of 1.3x10^4^ PFU/μg RNA which was only slightly lower than the infectivity of transcripts from wild type pToto1101 (4.0x10^4^ PFU/μg RNA). The infectivity of transcripts from pToto1101^ΔT^ was strongly reduced (1.5x10^3^ PFU/μg RNA) and that of transcripts from pToto1101^css^ was close to the limit of detection of the assay (<20 PFU/μg RNA). Thus, the infectivity of transcripts correlated, at least in terms of rank order, with the observed replication efficiencies of the corresponding templates in the *trans*-replication assay (Fig 10B and 10C). Furthermore, the difference observed between transcripts of pToto1101^ΔT^ and these of pToto1101^css^ may reflect the complexity of reversions/adaptations. For pToto1101^ΔT^ transcripts, an addition of a single U residue to the 5’ end of the RNA will restore its length and generate wild type like sequence. Addition of A and U residues to the ends of imperfect genomes is an adaptation commonly found in alphaviruses [58]. In contrast, in order to increase replication of the css-S-S template at least three insertions, none of which had major effect on its own, were required (Fig 10C). Apparently, re-creating the recovery pathway–or, more likely, creating one of its own–was more time consuming and this was reflected in low infectivity of corresponding transcripts. Whatever solution(s) were developed by mutant viruses they were clearly effective as all recombinant viruses grew to high final titres (>2.5x10^8^ PFU/ml) that were similar to these of wild type SINV. No correlation between the final titres and the efficiency of the corresponding template replication was observed.

## Discussion

*Trans-*replicases were developed for nine alphaviruses across the *Alphavirus* genus. Compared with actual viruses these represent safe and easy-to-use systems. *Trans-*replicases of viruses of different origin can be used in the same cell types and the relative ease of quantifying reporter enzyme activities, compared with viral RNA levels, allows use of the system for studies that generate large panels of data, such as screening for antiviral compounds or for host factors essential for virus replication. The sensitivity of the *trans*-replication system depends on the amplitude of “boost” of marker expression. The “boost” was always larger for transcription marker (Gluc) expression and, in general, showed an excellent correlation with the actual SG RNA production (Figs 2, 3 and 6). Nevertheless, it should be noted that the RNA/marker protein ratio is susceptible to the virus-induced shutoff of cellular transcription and translation that may be captured by *trans*-replicases or be host cell type specific. Compared to Gluc, the boost of the replication marker (Fluc) was almost always smaller. In human cells, however, it was prominent enough to serve as proxy of viral genomic RNA synthesis (Fig 6). In mosquito cells the boost of Fluc is low or modest and its correlation with genomic RNA synthesis is less pronounced (Fig 3) generally hampering comparison of different viruses. It is, however, still useful for comparison of the effects of mutations introduced into replicase of a specific alphavirus [45]. Furthermore, the low boost of Fluc expression makes it useful for analysis of mutations or other effects that increase replication efficiency [59]. In this study several heterologous replicase/template combinations that resulted in increased RNA replication in mosquito cells were revealed: RRV replicase replicated MAYV template ~90-fold more efficiently and MAYV replicase replicated EILV template ~40-fold more efficiently than their own templates (S6 Fig). Accordingly, this *trans-*replication assay could be applied for analysis of molecular mechanisms behind these effects. Taken together, certain limitations of *trans-*replication assay may, depending on circumstances, also be regarded as benefits.

The new tools were used to assess the compatibility of template RNAs and replicases of different alphaviruses in human and *Ae albopictus* cells. Our analysis of determinants allowing efficient use of template by SINV replicase (Figs 9 and 10) correlated well with data previously obtained using infectious virus [32] or alphavirus replicons and DI RNA templates [26]. The high degree of correlation indicates that the alphavirus *trans*-replication system is not only useful for analysis of alphavirus proteins [45,60,61] and to study the importance of host factors [44,62] but is also fully suitable to study the interactions between alphavirus replicases and template RNAs. Several previously unknown properties, revealed in this study, considerably expand our understanding regarding the alphavirus replication process and may provide novel insight in the phylogeny of the genus.

One of the key findings in this study is that replicases of alphaviruses from the SFV complex can use RNA templates from each other as well as those from outgroup viruses. We recently demonstrated that SL structures located in the 5' region of the CHIKV template RNA are essential for genome replication, indicating that they are recognized by components of CHIKV replicase complex [33]. The determinants of sequence specificity for viruses from the SFV complex are also located at the 5' region of the template RNA. The determinants of RRV are preferentially recognized by the RRV replicase and reduce the utilization of the corresponding template by the CHIKV replicase, while the determinants located at the 5' region of the CHIKV template have an opposite effect. Interestingly, SL structures located in 5' regions both CHIKV and RRV are predicted to be extremely homologous (S1 Fig), suggesting that determinants of template selectivity for these replicases are most likely associated with primary RNA sequence rather than secondary structure. The situation with template RNAs of outgroup viruses is somewhat different: the SINV template accommodates both SINV and CHIKV replicases. The secondary structures at the 5’ ends of genomes of outgroup viruses were predicted (S8 Fig) to be thermodynamically less stable (from -28.9 to -50.0 kJ/mol) than those at the 5’ ends of genomes of viruses from SFV complex (from -53.1 to -75.3 kJ/mol). However, no obvious correlation was observed between the capacity of replicase to use a heterologous template and stability of the secondary structure at its 5’ end (S4 Fig, S5 Fig, S8 Fig). Interestingly, small changes in 5’ end of SINV template RNA could dramatically reduce its use by the homologous replicase even in the absence of an impact on the stability of secondary structures (S8 Fig). Furthermore, the predicted secondary structure of the 5' extreme of S-S-S and c^T/TATT/14^ss-S-S template RNAs (that are efficiently used by SINV replicase) are similar to those of their inefficiently used counterparts, S^ΔT^-S-S and c^T/14^ss-S-S (S8 Fig). This raises the possibility that the determinants required for use as template RNAs in general are different from those required for recognition by specific replicases. Evidence suggests that the former acts via a mechanism involving multiple RNA secondary structure elements, while the latter is likely based on RNA length and/or primary sequence.

Another interesting, yet still unanswered, question is whether the ability of alphaviruses to use different templates represents a biological advantage or a restriction. We have previously demonstrated that the replicase of SFV can use cellular RNAs in order to generate double-stranded RNAs that are recognized by RIG-I and induce expression of type-I interferons [63]. The efficiency of synthesis of such RNAs is different for different alphaviruses, being much higher for the SFV and SINV replicases than for the replicase of RRV [64]. Thus, the ability of the replicase to synthesize such interferon-inducing RNAs does not correlate with the ability of the replicases of these viruses to use heterologous templates (Figs 4, 5 and 6). Furthermore, experiments with the SAV-template clearly demonstrate that the ability of alphavirus replicases to use heterologous RNAs as templates has its limits. CHIKV and MAYV have been shown to be able to co-infect mosquitoes, at least in the laboratory [65]. Co-infection of mosquitoes by different arboviruses creates the possibility of co-transmission from mosquito to human [66]. Co-infection can also occur if humans are bitten by mosquitoes carrying different alphaviruses. A study from Kenya revealed that 38% of people who had been exposed to ONNV and/or CHIKV have, in fact, been exposed to both of them [67]. Given the similarity between these viruses and findings that a CHIKV vaccine (and, presumably, CHIKV infection) confers protection against ONNV [2,68] it seems plausible that many of these people have been co-infected by these viruses. The ability of alphaviruses to cross-utilize each other’s templates, both in human and mosquito cells, can facilitate recombination between virus genomes, ensuring that resulting recombinant RNA genomes can be used by replicase(s) of parental viruses or by chimeric replicase created as a result of the recombination. Thus, while the benefits from the ability to cross-utilize templates of other alphaviruses are not obvious for an individual infection event, the ability may represent a potential factor for alphavirus evolution and may be the key for successful recombination between distantly related members of the genus. Cross-utilization of templates in mosquito cells, coupled with the promiscuous nature of template RNA of mosquito-specific alphaviruses, creates the possibility of recombination between divergent alphaviruses. This may include recombination between mosquito-specific and arbovirus members of the genus, potentially leading to novel viruses of either type. The increase of our knowledge about mosquito-specific alphavirus genomes may reveal if such recombination events have occurred and what type of viruses originated from such events.

The ability of the alphavirus replicase to specifically recognize its own 5' UTR and SG promoter elements in the template RNA indicates that some component(s) of the virus replicase are capable of recognizing primary sequence motifs and/or secondary structures. The helicase region of CHIKV nsP2 has been co-crystallized with an RNA oligonucleotide corresponding to the 3' end of the CHIKV genome [61]. However the interaction of this protein with RNA is not sequence-specific and, based on our data (Fig 9), alphavirus replicases do not differentiate between each other’s 3' UTR sequences. Specific interactions with sequences corresponding to both UTR regions and the SG promoter have been reported for the central domain of nsP3 [69]. Nevertheless, the strongest candidate as a specificity factor for template recognition is nsP4, the RNA polymerase subunit of the replicase. Its ability to bind viral RNAs is dependent on other ns-proteins but clearly occurs in a very specific manner, i.e. it is possible to block the interaction of nsP4 with the SG promoter without affecting its interaction with the genomic promoter [29,30]. Unfortunately, recombinant nsP4 of CHIKV has very low solubility and activity [70], which complicates *in vitro* analysis of nsP4-RNA interactions. It is also of great interest to reveal which RNA elements–sequences, secondary structures and/or long-range interactions—are crucial for specific template recognition. As alphavirus RNA can adopt alternative secondary structures [33], which most likely change in response to the interaction with different host and viral components, advantage could be taken of novel methods, allowing analysis of RNA structures and RNA-RNA interactions inside the cells. For such analysis, the *trans*-replicase system allows manipulation of sequence and secondary structures of template RNAs in a more efficient way than using infectious virus genomes.

It was observed that the *trans*-replicase of ONNV has low activity in human cells. It is possible that the poor activity of its *trans-*replicase may reflect a natural property of ONNV, or at least of the ONNV isolate used in this study. Interestingly, ONNV replicates reasonably well in C6/36 cells, a property that contrasts sharply with a near complete lack of activity of its *trans*-replicase in these cells. We have previously observed such a combination for *trans*-replicase/infectious virus of CHIKV harbouring mutations accelerating processing of P123 polyprotein precursor [59]. Most likely these findings are linked and represent a consequence of an additional hurdle for the formation of functional replicase complexes by *trans*-replicase—compared to those of infectious virus. Here we have revealed that such a hurdle, likely involving finding and binding template RNA provided *in trans*, has a much more prominent effect in *Ae albopictus* than in human cells (Fig 3D). Interestingly, alterations in the speed of P123 processing also has a much stronger impact on the *trans*-replicase activity in mosquito cells than in human cells [59]. The cell type specificity of this effect strongly argues for involvement of cell-specific conditions and/or cell-specific factors. Thus far the only known host factor present in both human and mosquito cells and absolutely required for the replication of CHIKV and ONNV is G3BP and its mosquito ortholog called Rasputin [44]. These proteins interact with the C-terminal region of nsP3 of Old World alphaviruses [71,72]. Interestingly, a CHIKV *trans*-replicase harbouring mutant nsP3 which is unable to interact with Rasputin, is virtually inactive in C6/36 cells [44] and therefore similar to the *trans*-replicase of ONNV. However, ONNV nsP3 does have sites for interaction with Rasputin and ONNV is competent for replication in mammalian cells that express G3BP. It is plausible that ONNV replicase can bind *Ae albopictus* Rasputin but uses it efficiently only in the context of infectious virus and not in the context of the *trans*-replicase. Thus, the replication defect, albeit detected in an artificial system, may actually be connected to the most important *in vivo* property of ONNV i.e. its transmission by *Anopheles* mosquitoes. Interestingly, the determinant of infection rates in *Anopheles gambiae* has also been mapped to nsP3 of ONNV [51]. Thus, this study adds another important piece of information to the puzzle regarding alphavirus nsP3, host G3BPs/Rasputin proteins and the mechanism(s) surrounding the formation and functioning of virus replicase complexes and ultimately its impact on alphavirus vector transmission. *Trans*-replicase systems, that reproduce, and possibly even emphasise, natural properties of alphavirus replicases, represent a valuable tool for studying such interactions.

Interesting findings were also obtained for EILV. Firstly, in sharp contrast to the template RNA of fish-infecting SAV, the template of mosquito-specific EILV was efficiently used by replicases of arbovirus members of alphaviruses in both mosquito and human cells. At the same time, EILV replicase was highly specific to its own template RNA. Both of these properties are similar to SINV, furthermore replicases of both viruses were able to use each other’s templates better than any other heterologous template. It is plausible that these functional similarities originate from the phylogenetic relationship between these viruses (Fig 1C) and similar folding of 5’ regions of these template RNAs (S1 Fig). This raises an intriguing question: did a SINV-like ancestor lose its ability to replicate in vertebrates or did an EILV-like ancestor acquire the ability to replicate in a vertebrate host? The number of recognized insect-specific alphaviruses is rapidly increasing and the same is the case for other virus groups traditionally considered as arboviruses. Given that insect-specific viruses from these groups are widespread and abundant, they may represent ancestors of arbovirus members of corresponding groups. Here we show that, in order to become an arbovirus, they do not need to acquire the ability of replicating in mammalian cells: this activity already exists—at least for EILV (Fig 2). Increased replication efficiency and adaptation to elevated temperatures are effects frequently observed for alphavirus temperature sensitive mutants propagated in cell culture; consequently, it seems plausible that insect-specific alphaviruses have, perhaps repeatedly, adapted to infection of vertebrate hosts. Thus, *trans*-replicase assays are versatile tools to examine the poorly understood relationship between insect-specific viruses and arboviruses that cause infections in humans.

As template switching by the viral replicase represents the most efficient mechanism for recombination between viruses, the potential for cross-utilization of templates could shed light on the mechanisms of (alpha)virus recombination and evolution. The *trans-*replication approach developed here is applicable to other virus families containing major human pathogens and/or emerging viruses such as *Picornaviridae*, *Flaviviridae* and *Coronaviridae*. *Trans*-replicases are flexible, easy to use systems that offer an alternative to study viral recombination and evolution when infectious clones are not available. Their use also eliminates the need for high containment facilities and allows convenient assessment of template cross-utilization, and thus potential for recombination, without the need for creating recombinant viruses in the lab.

Better understanding of the template RNA requirements of (alpha)virus replicases creates numerous possibilities. Promiscuous templates can be used for analysis of interactions between replicase proteins of (alpha)viruses, RNA-replicase interactions and for reconstruction of (alpha)virus replication complexes. Sub-optimal combinations of 5' UTR sequences and replicases can be used to generate recombinant attenuated viruses with potential use as vaccine candidates. The *trans*-replicases of alphaviruses can be used as tools for inducible expression of proteins of interest. The use of replicases from Old World alphaviruses may be hampered by the cytotoxic effects of their nsPs. However, here we demonstrate that the less cytotoxic replicase of VEEV produces a comparable boost in protein expression via activity of the SG promoter (Fig 2). This property, likely shared by replicases of other New World alphaviruses, may be utilized for development of efficient expression systems with extremely high induced/uninduced expression ratios (i.e. ratio of expression levels with and without replicase). Furthermore, constructs expressing promiscuous templates can be used to obtain transgenic cell lines that can serve as universal biosensors of alphaviruses. This approach may be applied to the detection and identification of unknown alphaviruses, especially those belonging to the SFV complex and capable of using different RNA templates. Other virus families with *trans*-active replicases may take advantage of this technology. It may even be possible to extend this approach to transgenic mosquitoes, potentially providing rapid, robust biosensors not dependent on access to tissue culture facilities, and perhaps providing a new route to develop methods to modify wild vector populations with specific, engineered response elements to arboviruses allowing detection and/or reduced vector competence. Conversely, alphavirus group-specific templates and SG promoters can be utilized in biosensors that allow discrimination between alphaviruses belonging to different serocomplexes and potentially even between different species of alphaviruses. Finally, the critical sequences and RNA secondary structures required for replication are interesting new and potentially specific targets for antiviral interference with molecules such as locked nucleic acids and morpholino oligonucleotides.

## Methods

### Cells and viruses

U2OS human bone osteosarcoma cells (ATCC HTB-96) were maintained in Iscove's modified Dulbecco's medium (Gibco) containing 10% fetal bovine serum (FBS) and 2 mM L-glutamine at 37°C in a 5% CO_2_ atmosphere. HEK293T cells (ATCC CRL-3216) were maintained in Dulbecco's modified Eagle medium (DMEM) with 2mM L-glutamine and 10% FBS at 37°C in a 5% CO_2_ atmosphere. BHK-21 cells (ATCC CCL-10) were grown in Glasgow's minimal essential medium (Gibco) containing 10% FBS, 2% tryptose phosphate broth (TPB) and 200 mM HEPES pH 7.2 at 37°C in a 5% CO_2_ atmosphere. *Aedes albopictus* derived C6/36 cells were maintained in Eagle's minimum essential medium containing 10% FBS at 28°C with no extra CO_2_. All media were supplemented with 100 U/mL penicillin and 0.1 mg/mL streptomycin.

### Plasmids

Plasmids containing native (not codon optimized) sequences encoding for P1234 of CHIKV (ECSA genotype, LR2006-OPY1 isolate) or its polymerase negative variant P1234^GAA^, harboring GDD to GAA substitution in the active site of nsP4, under the control of immediately early promoter of human cytomegalovirus (CMV) have been previously described [43]. Similar plasmids designed for the expression of P1234 of SFV (SFV4 strain), SINV (Toto1101 strain), RRV (RRV-T48 strain), MAYV (TRVL strain), ONNV (Chad isolate), BFV and VEEV (V3526 strain) as well as their polymerase negative variants in mammalian cells have also been described [44]. The plasmid for expression of P1234 of EILV in mammalian cells has similar design. It was assembled from synthetic DNAs (GenScript, USA) and restriction fragments of corresponding infectious cDNA clone which was a gift from Scott Weaver (UTMB) [7]; its polymerase negative variant was obtained using site-directed mutagenesis and sub-cloning procedures. For simplicity, and in order to avoid confusion with each other, these plasmids were designated as CMV-P1234-CHIKV, CMV-P1234^GAA^-CHIKV and so on. To obtain constructs for expression of replicases in *Ae albopictus* cells, the P1234 encoding regions were cloned under the control of polyubiquitin promoter from *Aedes aegypti* as previously described for CHIKV *trans*-replicase [59]; obtained clones were designated as Ubi-P1234-CHIKV, Ubi-P1234^GAA^-CHIKV and so on.

Human RNA polymerase I promoter-based plasmids for the production of replication competent RNA templates of CHIKV (originally named HSPolI-Fluc-Gluc), SFV, ONNV, SINV, RRV, BFV and VEEV have been previously described [44,45]. The plasmids for expression of RNA templates of EILV and SAV had the same design and were assembled from synthetic DNA fragments (GenScript, USA). To obtain constructs for expression of corresponding template RNAs in *Ae albopictus* cells the design developed for CHIKV (originally named AlbPolI-Fluc-Gluc [45]), was adapted to all above listed alphaviruses except SAV. For simplicity these plasmids were designated as HSPolI-FG-CHIKV, AlbPolI-FG-CHIKV and so on. For detection of individual cells positive for RNA replication the *Gaussia* luciferase (Gluc) marker in HSPolI-FG-CHIKV and AlbPolI-FG-CHIKV was replaced with ZsGreen; resulting in vectors designated HSPolI-FZsG-CHIKV and AlbPolI-FZsG-CHIKV.

For making SINV/CHIKV swapped templates the region corresponding to the 5' end of CHIKV template RNA was extended from nucleotide 307 to nucleotide 412; obtained plasmid was designated as HSPolI-FG-C*CC. The swapping of 5' region (5' UTR+ region encoding for N-terminus of nsP1), SG promoter region and 3' region (truncated 3' UTR) between HSPolI-FG-C*CC and HSPolI-FG-SINV or HSPolI-FG-CHIKV and HSPolI-FG-RRV was performed using standard cloning procedures. Obtained constructs were designated as explained on Fig 9A, HSPolI-FG-SSS, HSPolI-FG-C*SS and so on. Corresponding template RNAs were designated as S-S-S, C*-S-S and so one. Note that plasmid designated as HSPolI-FG-CCC is the same as HSPolI-FG-CHIKV, HSPolI-FG-SSS is the same as HSPolI-FG-SINV and HSPolI-FG-RRR is the same as HSPolI-FG-RRV.

Plasmids for the expression of templates with changes in the 5' region were constructed using synthetic DNAs (GenScript, USA) and subcloning procedures. The first set of constructs contained swaps of SL3, SL47 regions and nsP1 ORF parts in template RNA encoded by HSPolI-FG-SSS (Fig 10A, left panel). The obtained plasmids were designated as HSPolI-FG-ssc*SS, HSPolI-FG-ccsSS and so on; the first small letter in the name of plasmid reflects the origin of the 5' extreme and SL3, second small letter reflects the origin of the region corresponding to CHIKV SL47; the third small letter reflects the origin of the region encoding the N-terminus of nsP1; c* indicates use of longer fragment of CHIKV nsP1 ORF. In the second set of plasmids the 5' ends and SL3 regions of templates encoded by HSPolI-FG-SSS and HSPolI-FG-cssSS were modified as shown on Fig 10A (right panel). Constructs based on HSPolI-FG-SSS were designated as HSPolI-FG-S^ΔT^SS, HSPolI-FG-S^ΔTATT^SS and HSPolI-FG-S^Δ14^SS; constructs based on HSPolI-FG-cssSS were designated as HSPolI-FG-c^T^ssSS, HSPolI-FG-c^TATT^ssSS, HSPolI-FG-c^14^ssSS, HSPolI-FG-c^T/14^ssSS and HSPolI-FG-c^T/TATT/14^ssSS.

Plasmid pToto1101 was used to construct infectious cDNA clones of SINV harbouring modifications in the 5' UTR. The deletion of a residue corresponding to the position 3 of SINV genome was performed using site-directed mutagenesis and subcloning procedures, corresponding plasmid was designated pToto1101^ΔT^. Replacements identical to these used in HSPolI-FG-cssSS and HSPolI-FG-c^T/TATT/14^ssSS template-RNA encoding plasmids were made using synthetic DNAs (GenScript) and subcloning procedures; obtained plasmids were designated pToto1101^css^ and pToto1101^cT/TATT/14ss^, respectively.

Sequences of all plasmids were verified using Sanger sequencing and are available from the authors upon request.

### Rescue of recombinant viruses

Virus rescue in BHK-21 cells and ICA were performed as previously described [73]. Briefly, BHK-21 (8×10^6^) were transfected with *in vitro* transcripts by electroporation with a Bio-Rad Gene Pulser II unit (two pulses at 850 V and 25 μF) in 0.4 cm cuvettes (Thermo Fisher Scientific). After 2 h of incubation at 37°C, the cells were overlaid with 2 ml of GMEM supplemented with 2% FBS and containing 1% Bacto^TM^ agar (BD Biosciences). For virus rescue experiments virus stocks were collected at 24 h (wild type and pToto1101^c+T/TATT/14ss^) or at 48 h (pToto1101^-T^ and pToto1101^css^) post transfection (p.t.). Obtained stocks were clarified by centrifugation at 3000xg for 10 minutes and virus titers were determined using standard plaque assay on BHK-21 cells.

### *Trans*-replication assay

The *trans*-replication assay in U2OS, HEK293T and C6/36 cells was performed as previously described [59]. In order to determine the optimal time point for analysis of reporter expression, the HEK293T and C6/36 cells were transfected with homologous pairs of plasmids (1 μg of plasmid encoding for template RNA and 1 μg of plasmid encoding for corresponding replicase) using Lipofectamine LTX (Thermo Fisher Scientific) according to the manufacturer’s instructions. Transfected cells were incubated at 37°C (HEK293T) or 28°C (C6/36). For HEK293 cells 10 μl aliquots of growth media were collected at 4, 8, 12, 16, 20, 22, 24 and 48 h p.t.; for C6/36 cells the samples were collected at 12, 24. 36, 48, 54, 66, 72 and 144 h p.t. The activity of secreted Gluc was measured using the *Renilla* luciferase assay system (Promega).

U2OS cells grown on 12-well plates were co-transfected with 1 μg of HSPolI-FG-CHIKV or other plasmid encoding for template RNA and 1 μg of CMV-P1234-CHIKV or other plasmid encoding for replicase; in control cells the later was replaced with plasmid encoding a polymerase negative version of replicase protein (CMV-P1234^GAA^-CHIKV and so on). Transfected cells were incubated at 37°C for 18 h. In experiments involving HSPolI-FG-EILV and CMV-P1234-EILV plasmids the transfected cells were also incubated at 28°C for 48 h. All experiments were repeated at least three times.

For HEK293T and C6/36 cells the *trans*-replication assay carried out using 96-well plate format. Briefly, approximately 35,000 cells per well were co-transfected with 0.05 μg of the template and 0.05 μg of appropriate replicase expression plasmids; in control cells the later was replaced with a plasmid expressing the polymerase negative version of replicase. Transfections were performed using Lipofectamine LTX reagent. HEK293T cells were generally incubated at 37°C for 18 h. HEK293T cells transfected using EILV cDNA derived plasmid as well as all transfected C6/36 cells were incubated at 28°C for 48 h. All transfections were performed in triplicate and experiments were repeated at least twice. After incubation, cells were lysed and firefly luciferase (Fluc) and Gluc activities were measured using the Dual-Luciferase-Reporter assay (Promega). Fluc and Gluc activities measured for cells transfected using plasmids expressing active replicases were normalized to these obtained for corresponding control cells.

### Flow cytometry assay

HEK293T cells grown in 12-well plates were co-transfected with 1 μg of HSPolI-FZsG-CHIKV and 1 μg of CMV-P1234-CHIKV; C6/36 cells grown in 12-well plates were co-transfected with 1 μg of AlbPolI-FZsG-CHIKV and 1 μg of Ubi-P1234-CHIKV. Control HEK293T cells were transfected with 1 μg of plasmid expressing EGFP from CMV promoter (CMV-EGFP) and control C6/36 cells were transfected with 1 μg of plasmid expressing EGFP from polyubiquitin promoter (Ubi-EGFP). Transfections were performed in triplicate using Lipofectamine LTX reagent. At 18 h (HEK293T) or 48 h (C6/36) p.t. cells were collected in 500 μL PBS and analyzed with an Attune NxT Acoustic Focusing Cytometer. For each sample 30,000 events were recorded. The acquired data was analyzed using Attune NxT software.

### Northern blotting

HEK293T and C6/36 cells grown in 12-well plates were co-transfected with 1 μg of template RNA expression plasmid and 1 μg of replicase expression plasmid using Lipofectamine LTX reagent; control cells were mock-transfected. At 18 h (HEK293T) or 48 h (C6/36) p.t. total RNA was extracted using TRIzol reagent (Life Technologies). 2 μg of total RNA was used for detection of positive strands and 10 μg of total RNA was used for detection of negative strands. RNAs were denatured for 10 min at 70°C in 2X RNA loading dye (Thermo Scientific), cooled on ice and separated on a denaturing gel (1% agarose/6% formaldehyde) using 1X MOPS buffer. RNA was transferred to a Hybond-N+ filter (GE Healthcare) and fixed using a UV Stratalinker 1800 (Stratagene). Digoxigenin (DIG)-labelled RNA probe complementary to residues 42–390 of the sequence encoding for Gluc marker was used to detect positive-strand RNAs; probe corresponding to residues 51–376 of the sequence encoding for Fluc marker was used to detect negative-strand RNAs. Filters were hybridized overnight; blots were washed and developed according to the manufacturer's (Roche) protocols.

### *In silico* analysis of RNA structure

*In silico* thermodynamic RNA structure and free energy predictions were carried out using UNAFOLD at 37°C on default settings (version 3.4) [74]. RNA structures were visualised using the VARNA software package [75].

### Statistical analysis

Statistical analysis was performed using GraphPad Prism software. Data were analyzed using Student’s unpaired one tailed t-test. P values of ≤0.05 (*), ≤0.01 (**), ≤0.001 (***) and ≤0.0001 (‘***) were used to represent degrees of significance for each mutant compared to wild-type. Each experiment was repeated to gain a minimum of 3 independent biological repeats. Raw data used to generate graphs is presented in S1 Data.

## Supporting information

S1 Fig Comparative RNA folding predictions for 9 alphaviruses used in the study. *In silico* predicted UNAFOLD thermodynamic predictions for stable RNA structures within the 5' UTR and adjacent ORF1 encoding region for of CHIKV, SINV and a range of divergent alphaviruses.
(PDF)

S2 Fig Efficiency of CHIKV *trans*-replication in HEK293T cells. HEK293T cells were mock-transfected (A), transfected with CMV-EGFP plasmid (B), co-transfected with HSPolI-FZsG-CHIKV and CMV-P1234-CHIKV (C) or co-transfected with HSPolI-FZsG-CHIKV and CMV-P1234^GAA^-CHIKV (D). At 18 h p.t. cells were collected and analyzed with an Attune NxT Acoustic Focusing Cytometer. For each panel one image out of three showing flow blot of living cells (left), flow blot of EGFP/ZsGreen fluorescence in live cells (middle) and the gating used (right) are shown. (E) Combined data from three experiments showing live cells count, EGFP/ZsGreen positive cells count, the percentage of EGFP/ZsGreen positive cells from live cells and a fluorescence intensity in EGFP/ZsGreen positive cells (R1 mean). Data are presented as mean +SD.
(TIF)

S3 Fig Efficiency of CHIKV *trans*-replication in C6/36 cells. C6/36 cells were mock-transfected (A), transfected with Ubi-EGFP plasmid (B), co-transfected with AlbPolI-FZsG-CHIKV and Ubi-P1234-CHIKV (C) or co-transfected with AlbPolI-FZsG-CHIKV and Ubi-P1234^GAA^-CHIKV (D). At 48 h p.t. cells were collected and analyzed with an Attune NxT Acoustic Focusing Cytometer. Data are presented as described for S2 Fig.
(TIF)

S4 Fig **Comparison of capacities of replicases from SFV complex to replicate (left) and transcribe (right) different template RNAs in human cells.** Data is replotted from Figs 4 and 5. X-axis shows different templates; Y-axis shows percentage of activity of replicase on different templates; the activity on homologous template is taken as 100%.
(TIF)

S5 Fig **Comparison of capacities of replicases from outgroup alphaviruses to replicate (left) and transcribe (right) different template RNAs in human cells.** Data is replotted from Figs 4 and 5. X-axis shows different templates; Y-axis shows percentage of activity of replicase on different templates; the activity on homologous template is taken as 100%.
(TIF)

S6 Fig **Comparison of capacities of replicases from SFV complex to replicate (left) and transcribe (right) different template RNAs in *Aedes albopictus* C6/36 cells.** Data is replotted from Figs 7 and 8. X-axis shows different templates; Y-axis shows percentage of activity of replicase on different templates; the activity on homologous template is taken as 100%.
(TIF)

S7 Fig **Comparison of capacities of replicases from outgroup alphaviruses to replicate (left) and transcribe (right) different template RNAs in *Aedes albopictus* C6/36 cells.** Data is replotted from Figs 7 and 8. X-axis shows different templates; Y-axis shows percentage of activity of replicase on different templates; the activity on homologous template is taken as 100%.
(TIF)

S8 Fig Comparative RNA folding predictions for SL3 region of SINV, CHIKV, mutant recombinant constructs and other alphaviruses used in the study. I*n silico* predicted UNAFOLD thermodynamic predictions for stable RNA structures within the SL3 region of SINV (S-S-S), CHIKV (C-C-C), S^ΔT^-S-S, c^T/14^ss-S-S, c^T/TATT/^ss-S-S and a range of divergent alphaviruses.
(PDF)

S1 Data Raw data used to generate Figures and Supporting Figures. (XLSX)
