# Supplementary figures and images for: Cross-utilisation of template RNAs by alphavirus replicases
Source: PLoS Pathog. 2020 Sep 4;16(9):e1008825. doi: 10.1371/journal.ppat.1008825 (PMC7498090; doi:10.1371/journal.ppat.1008825)

S1 Fig

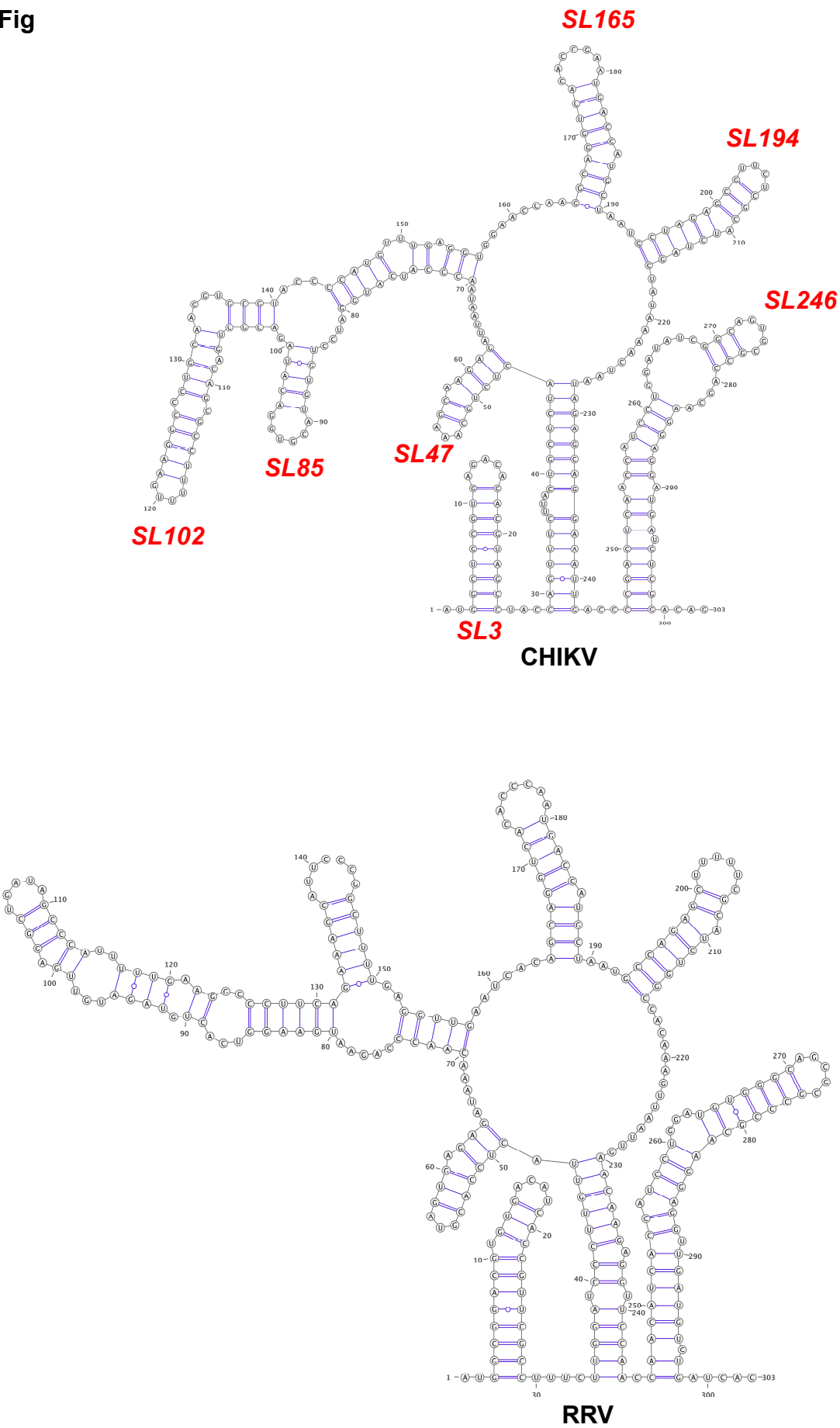

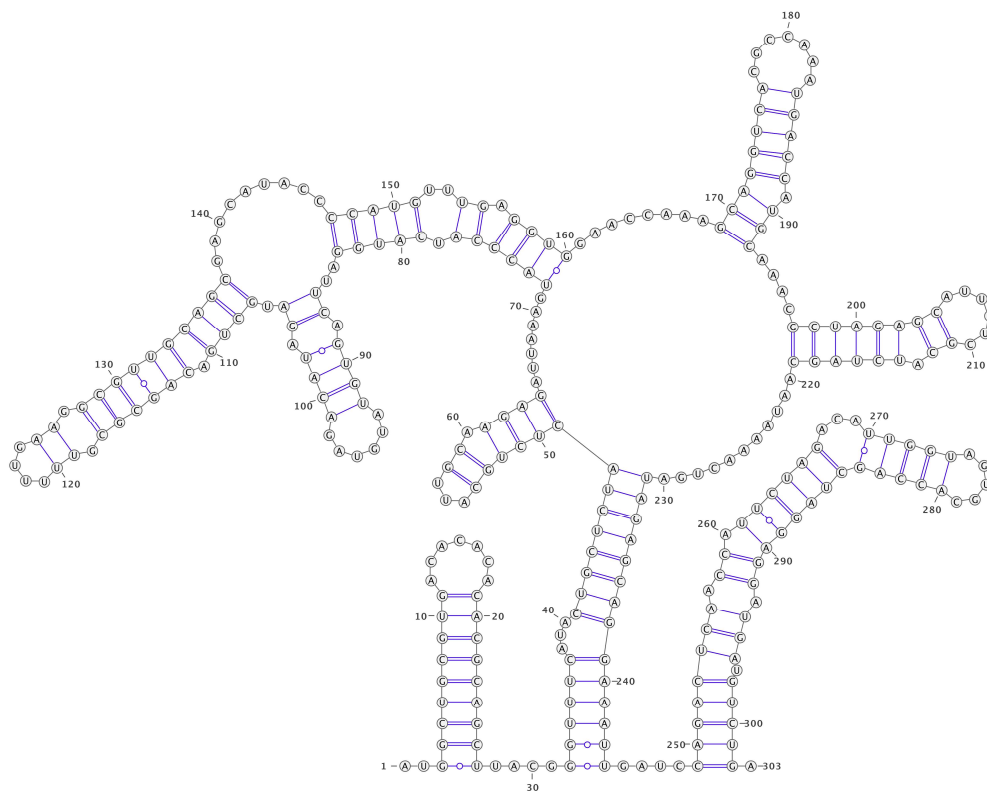

**ONNV**

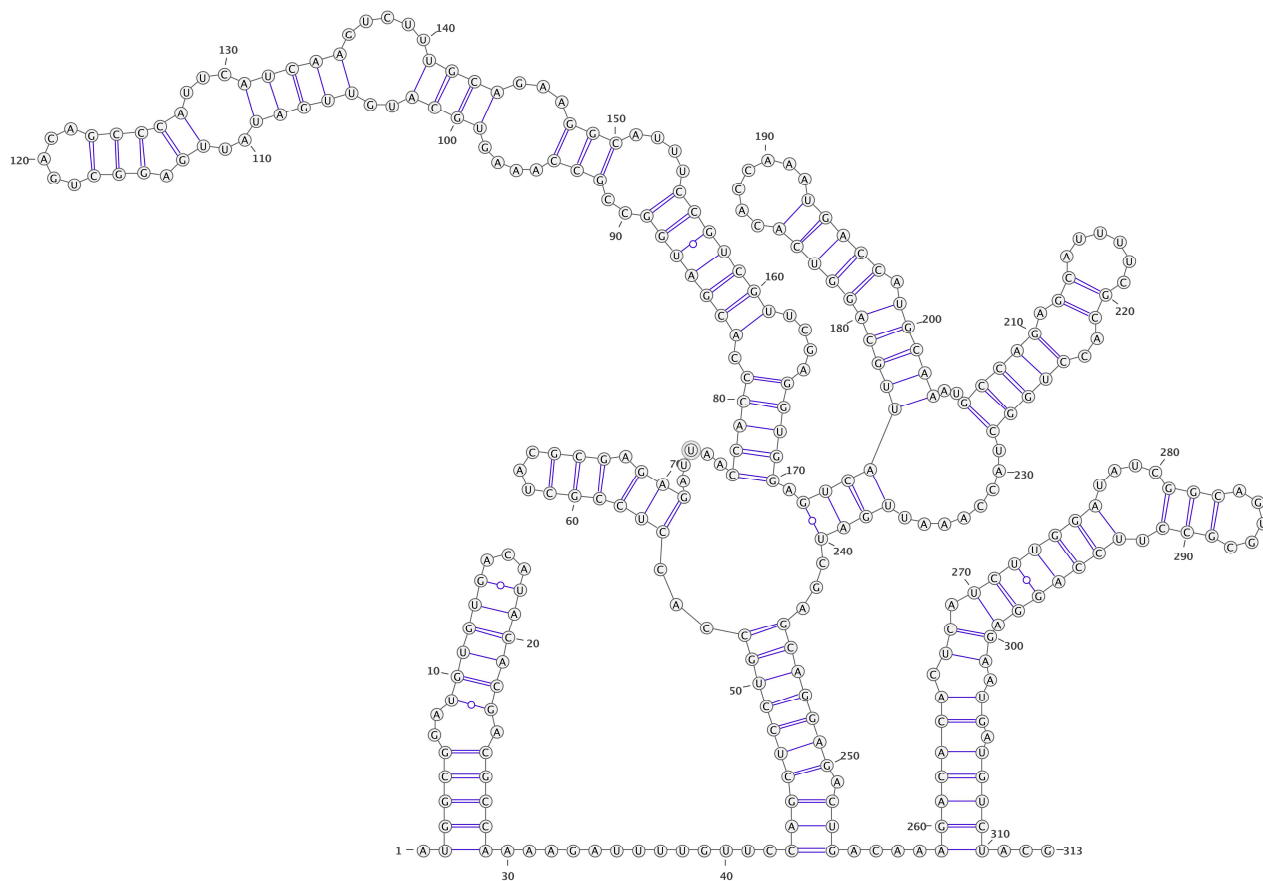

**SFV**

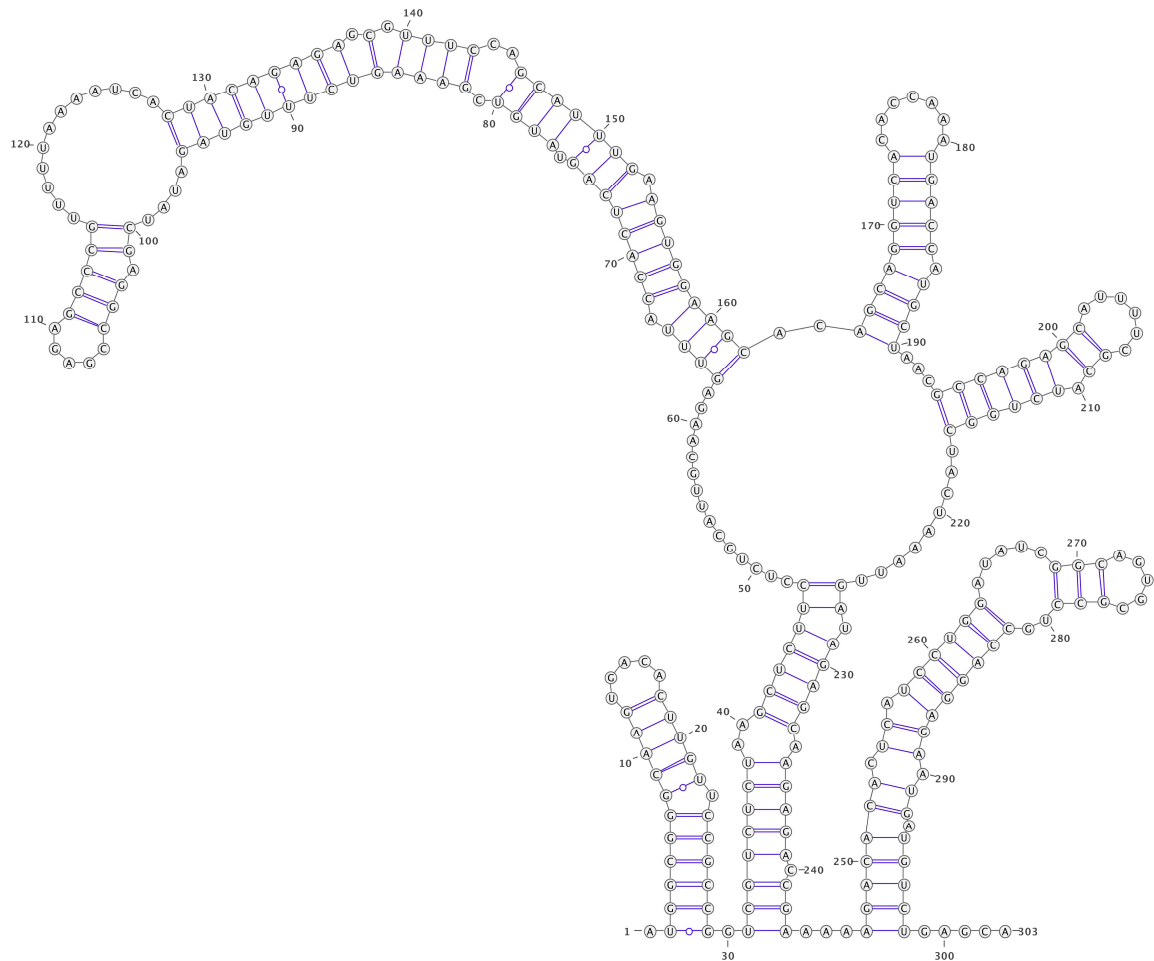

## MAYV

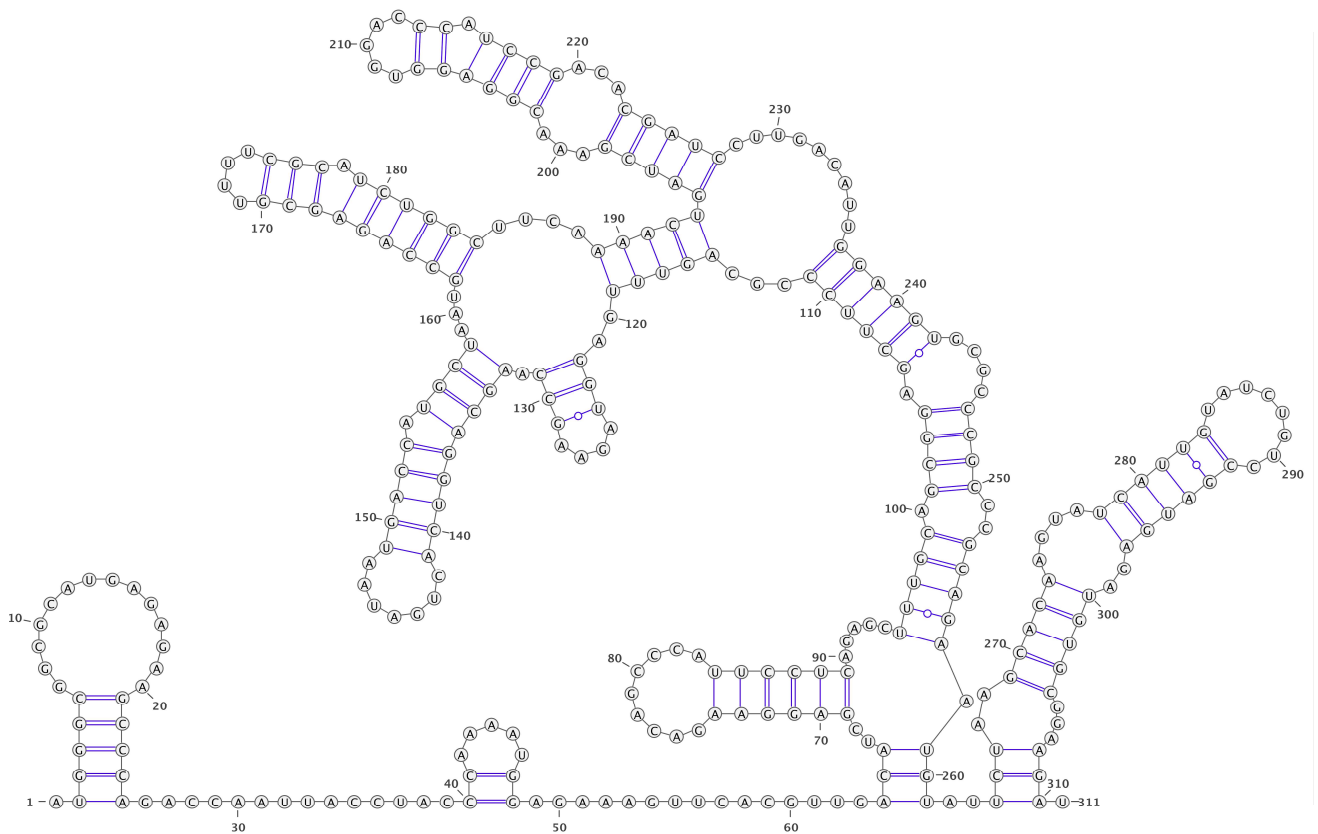

## VEEV

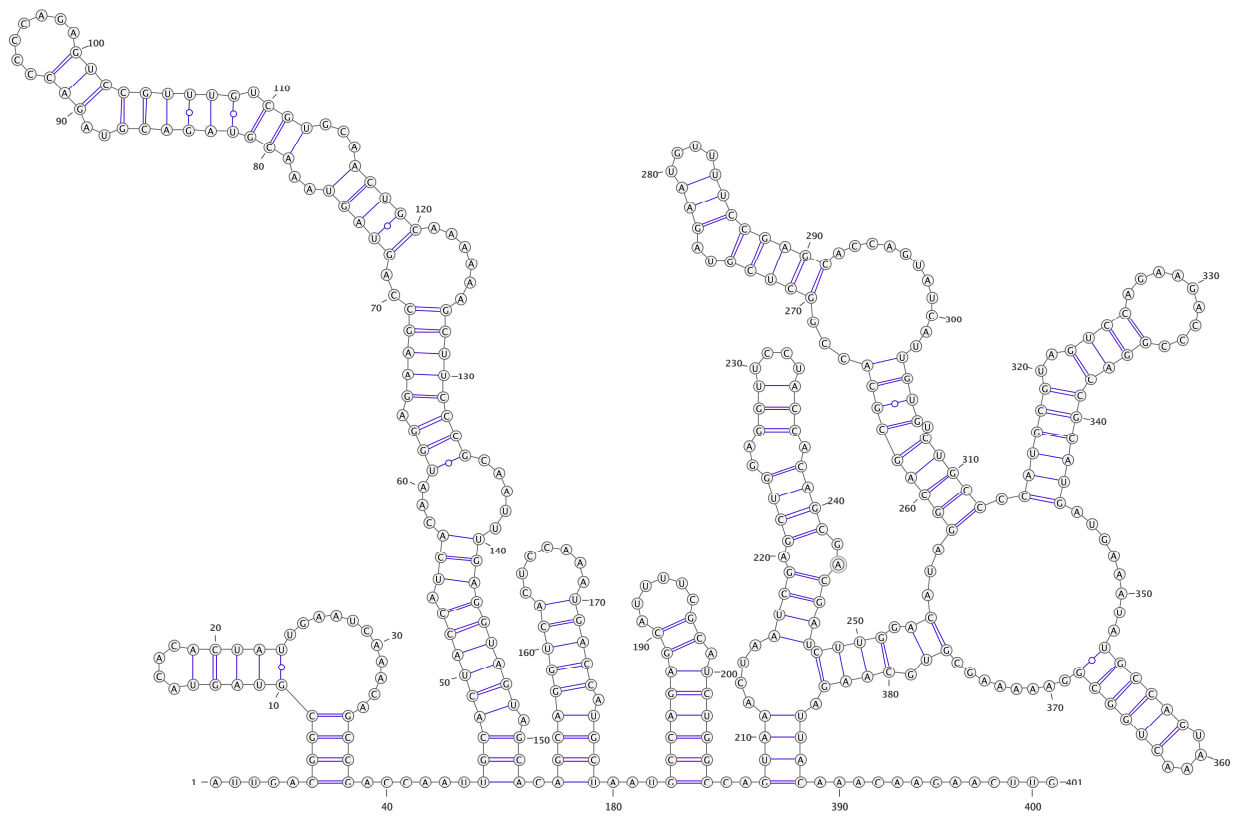

**SINV**

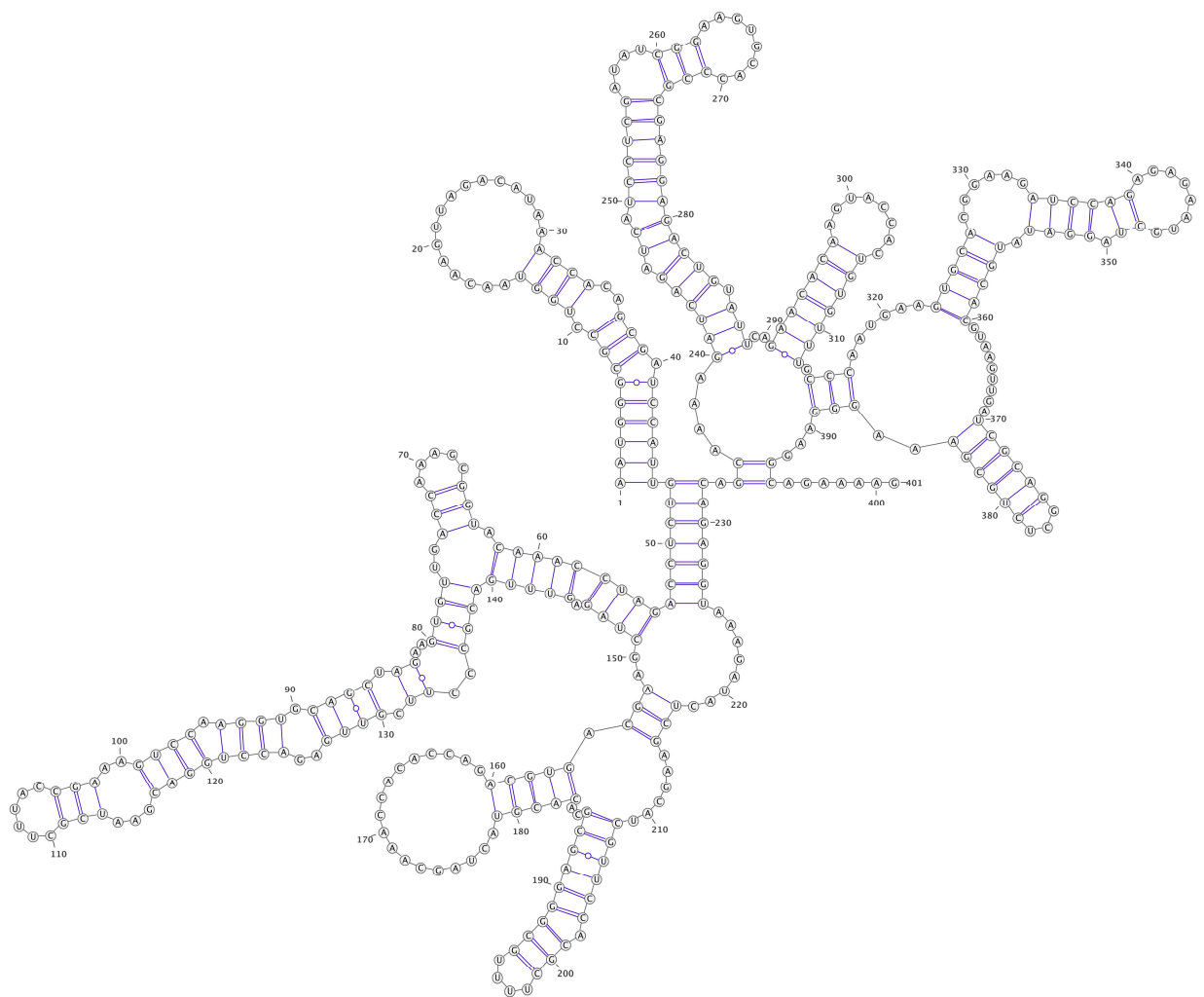

**BFV**

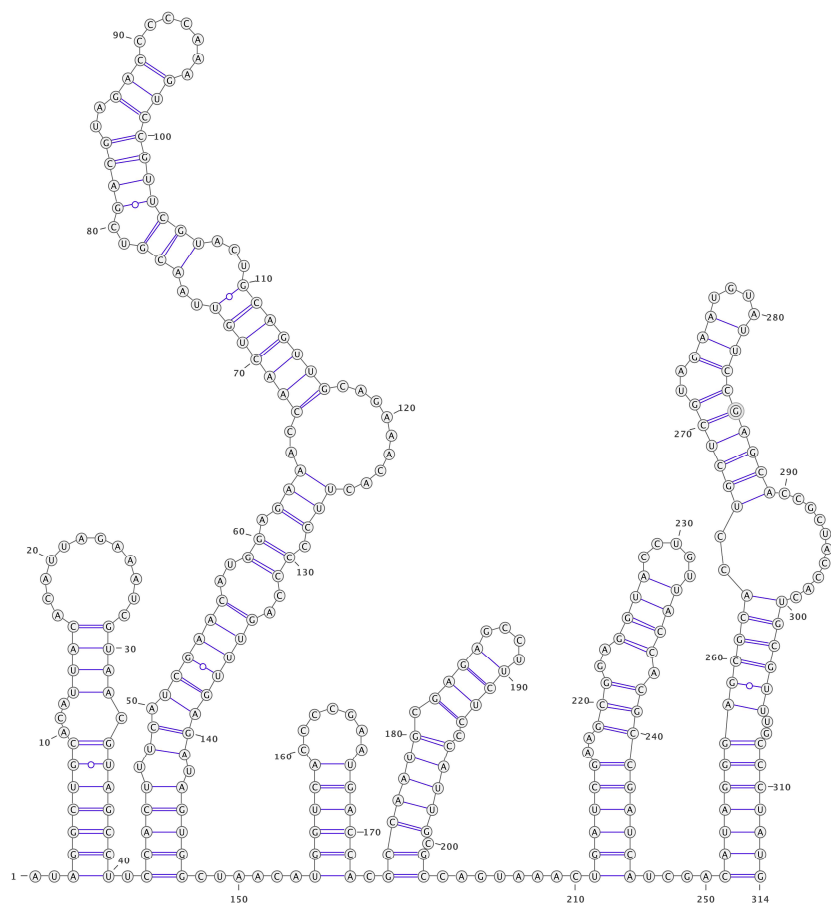

**EILV\_37°C**

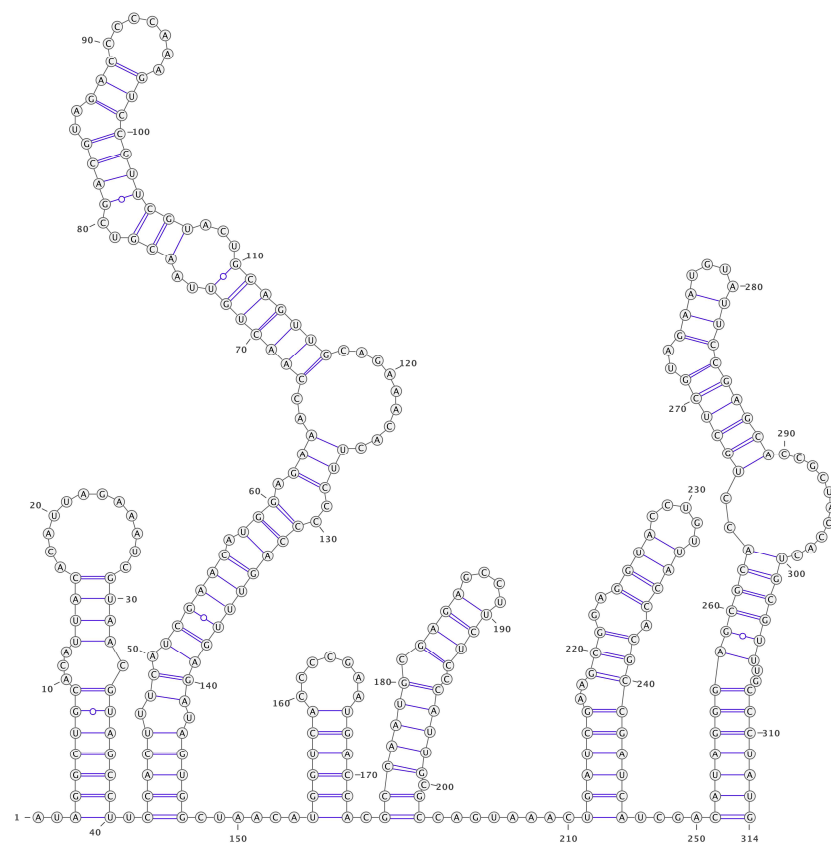

**EILV\_28°C**

Supplement: S1 Fig — In silico predicted UNAFOLD thermodynamic predictions for stable RNA structures within the 5' UTR and adjacent ORF1 encoding region for of CHIKV, SINV and a range of divergent alphaviruses. (PDF) [file ppat.1008825.s001.pdf]

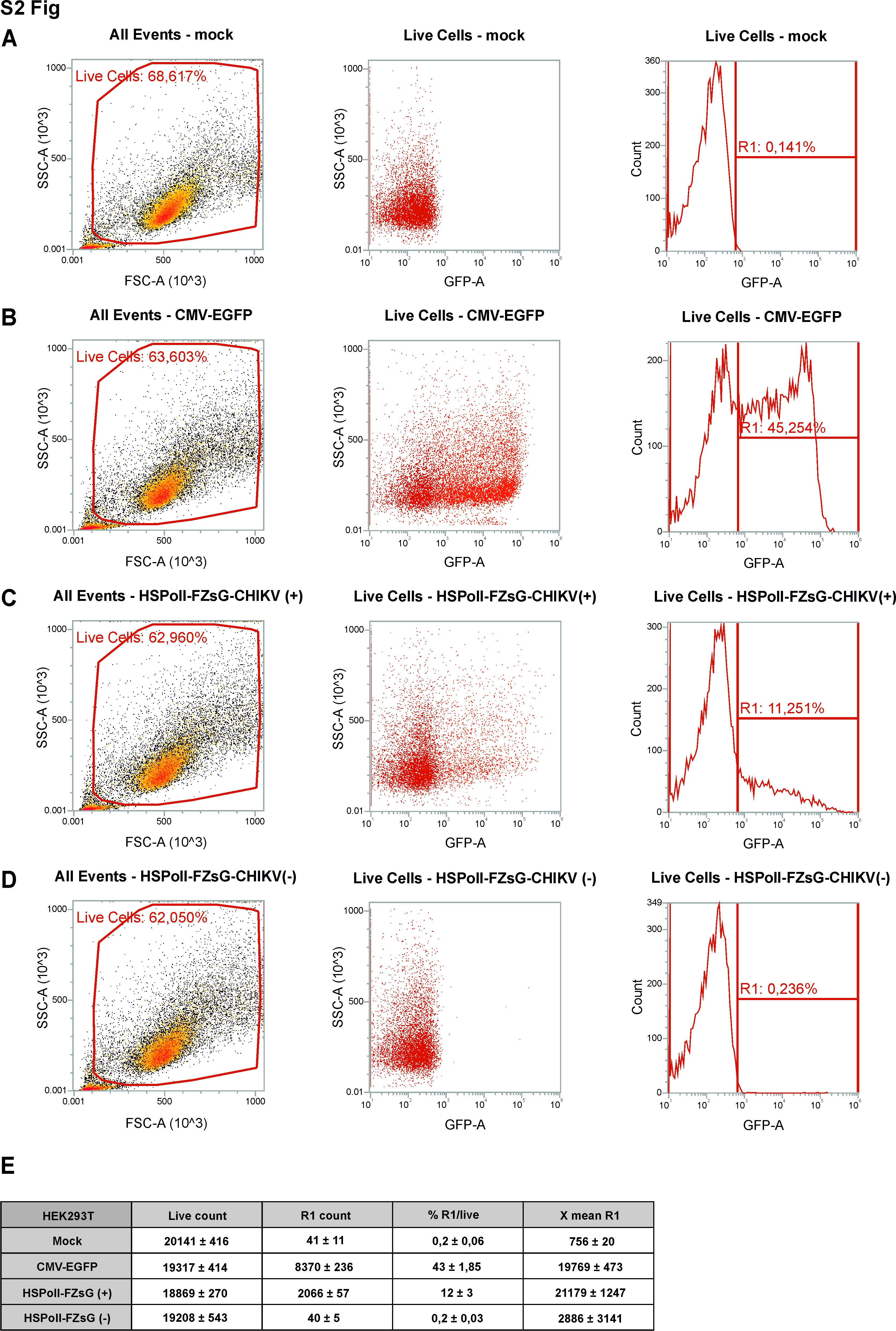

Supplement: S2 Fig — HEK293T cells were mock-transfected (A), transfected with CMV-EGFP plasmid (B), co-transfected with HSPolI-FZsG-CHIKV and CMV-P1234-CHIKV (C) or co-transfected with HSPolI-FZsG-CHIKV and CMV-P1234GAA-CHIKV (D). At 18 h p.t. cells were collected and analyzed with an Attune NxT Acoustic Focusing Cytometer. For each panel one image out of three showing flow blot of living cells (left), flow blot of EGFP/ZsGreen fluorescence in live cells (middle) and the gating used (right) are shown. (E) Combined data from three experiments showing live cells count, EGFP/ZsGreen positive cells count, the percentage of EGFP/ZsGreen positive cells from live cells and a fluorescence intensity in EGFP/ZsGreen positive cells (R1 mean). Data are presented as mean +SD. (TIF) [file ppat.1008825.s002.tif]

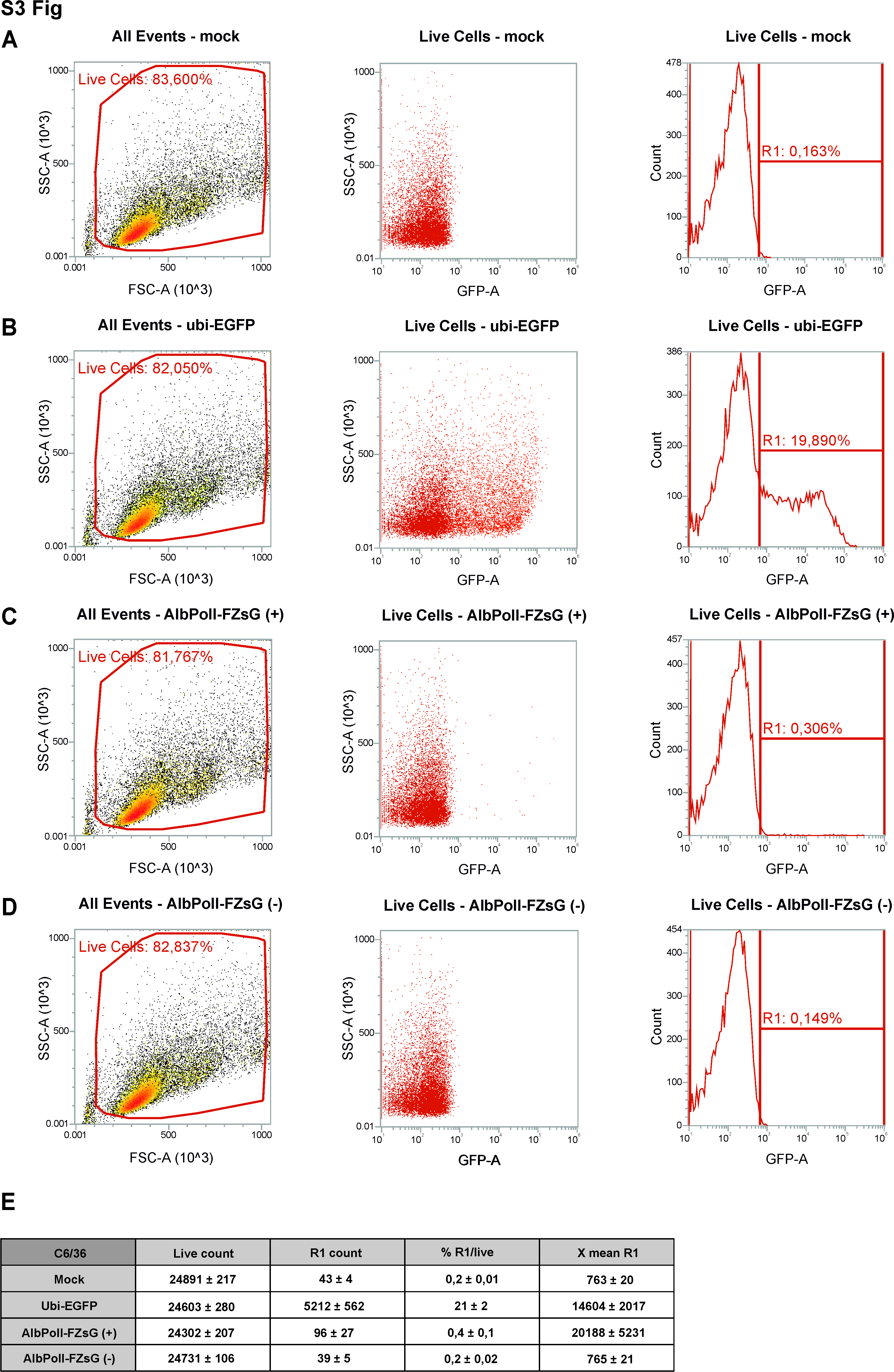

Supplement: S3 Fig — C6/36 cells were mock-transfected (A), transfected with Ubi-EGFP plasmid (B), co-transfected with AlbPolI-FZsG-CHIKV and Ubi-P1234-CHIKV (C) or co-transfected with AlbPolI-FZsG-CHIKV and Ubi-P1234GAA-CHIKV (D). At 48 h p.t. cells were collected and analyzed with an Attune NxT Acoustic Focusing Cytometer. Data are presented as described for S2 Fig. (TIF) [file ppat.1008825.s003.tif]

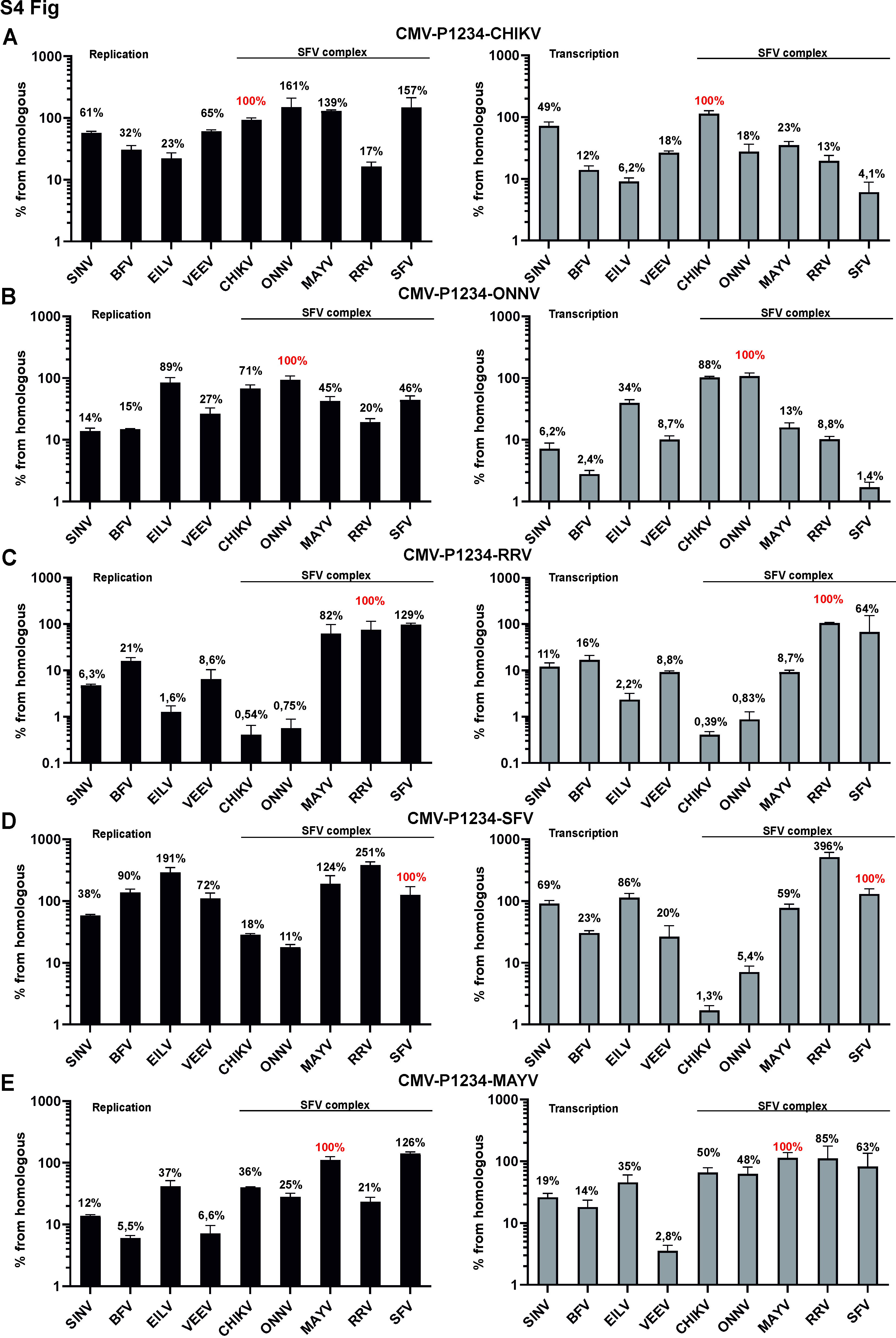

Supplement: S4 Fig — Comparison of capacities of replicases from SFV complex to replicate (left) and transcribe (right) different template RNAs in human cells. Data is replotted from Figs 4 and 5. X-axis shows different templates; Y-axis shows percentage of activity of replicase on different templates; the activity on homologous template is taken as 100%. (TIF) [file ppat.1008825.s004.tif]

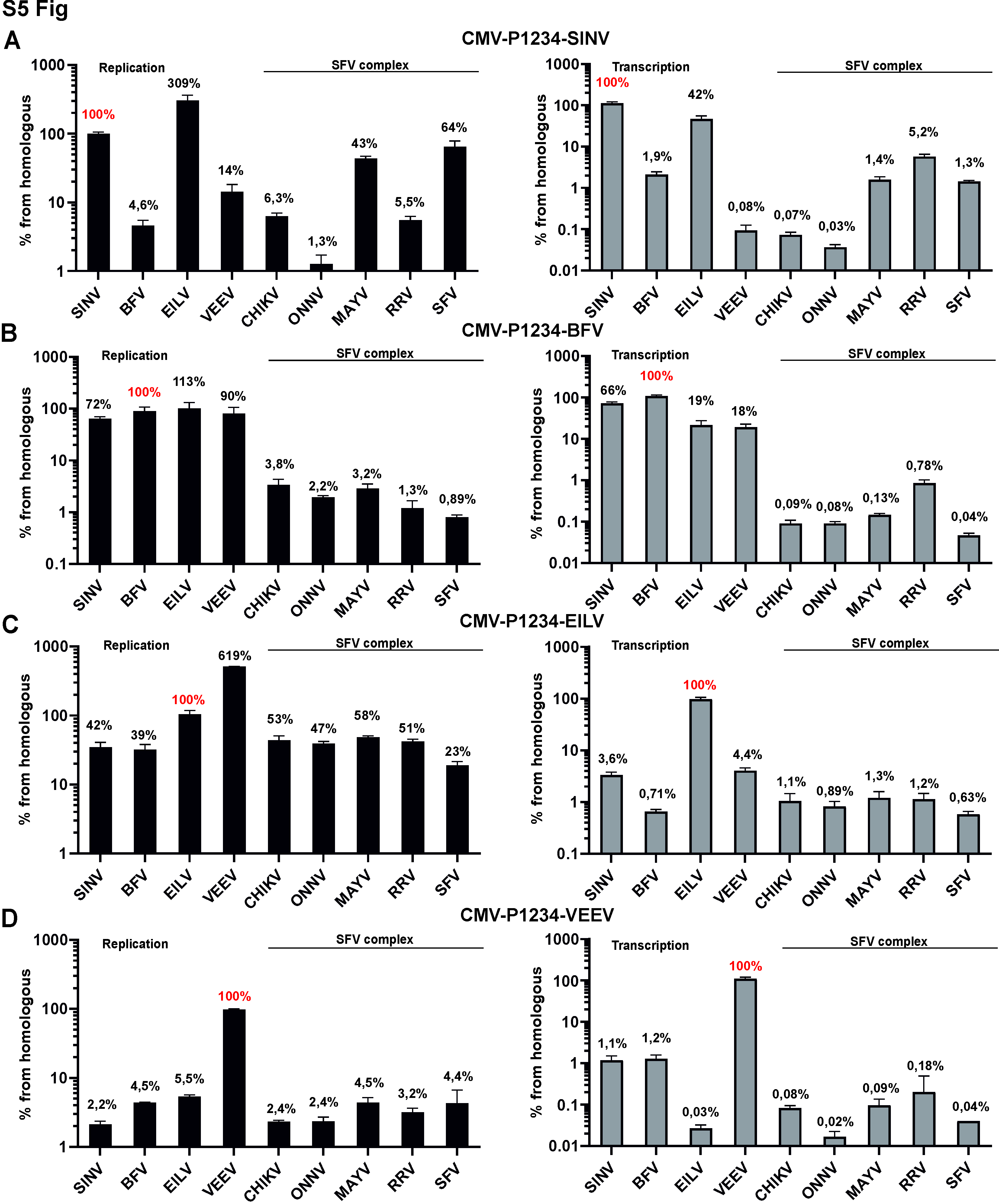

Supplement: S5 Fig — Comparison of capacities of replicases from outgroup alphaviruses to replicate (left) and transcribe (right) different template RNAs in human cells. Data is replotted from Figs 4 and 5. X-axis shows different templates; Y-axis shows percentage of activity of replicase on different templates; the activity on homologous template is taken as 100%. (TIF) [file ppat.1008825.s005.tif]

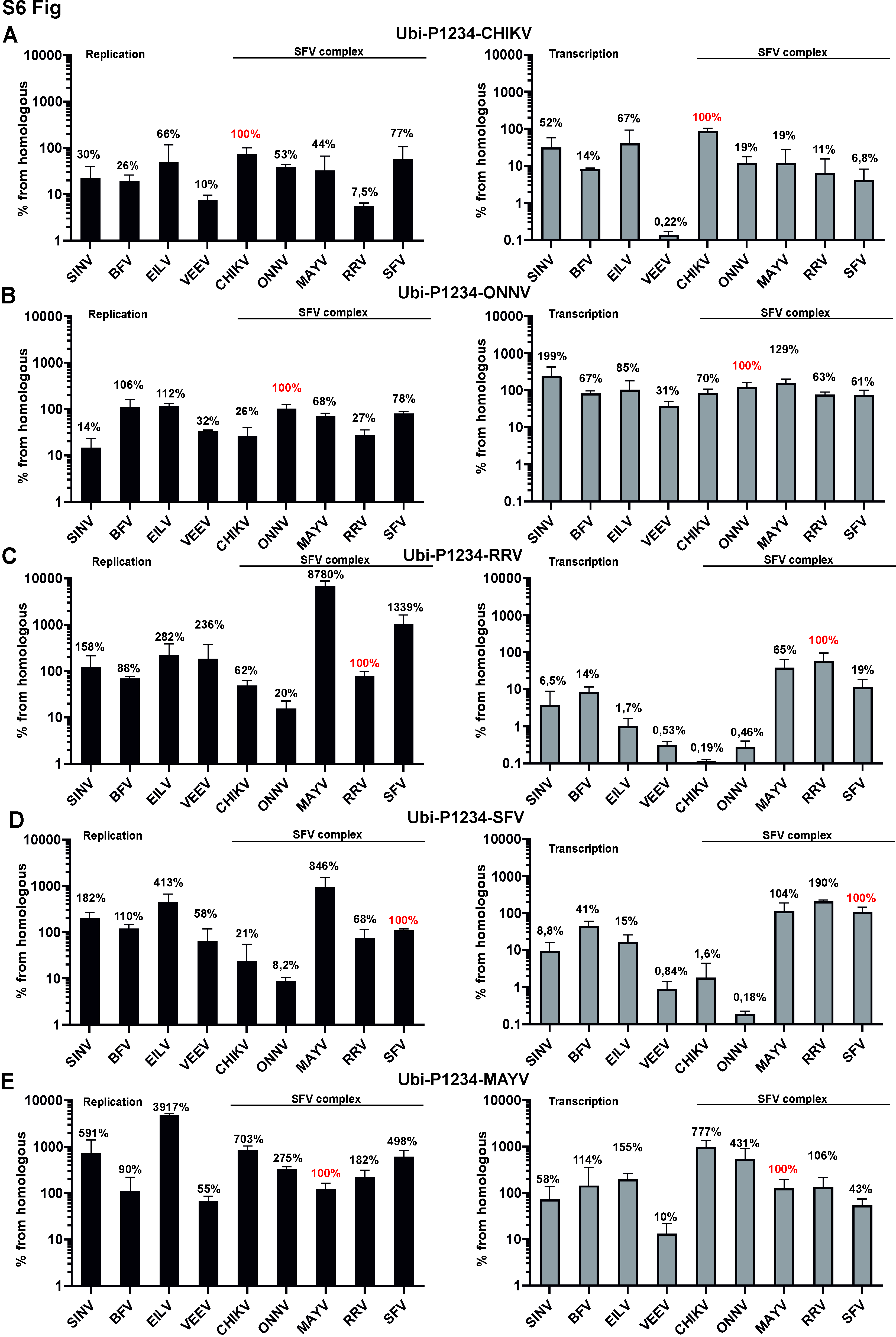

Supplement: S6 Fig — Comparison of capacities of replicases from SFV complex to replicate (left) and transcribe (right) different template RNAs in Aedes albopictus C6/36 cells. Data is replotted from Figs 7 and 8. X-axis shows different templates; Y-axis shows percentage of activity of replicase on different templates; the activity on homologous template is taken as 100%. (TIF) [file ppat.1008825.s006.tif]

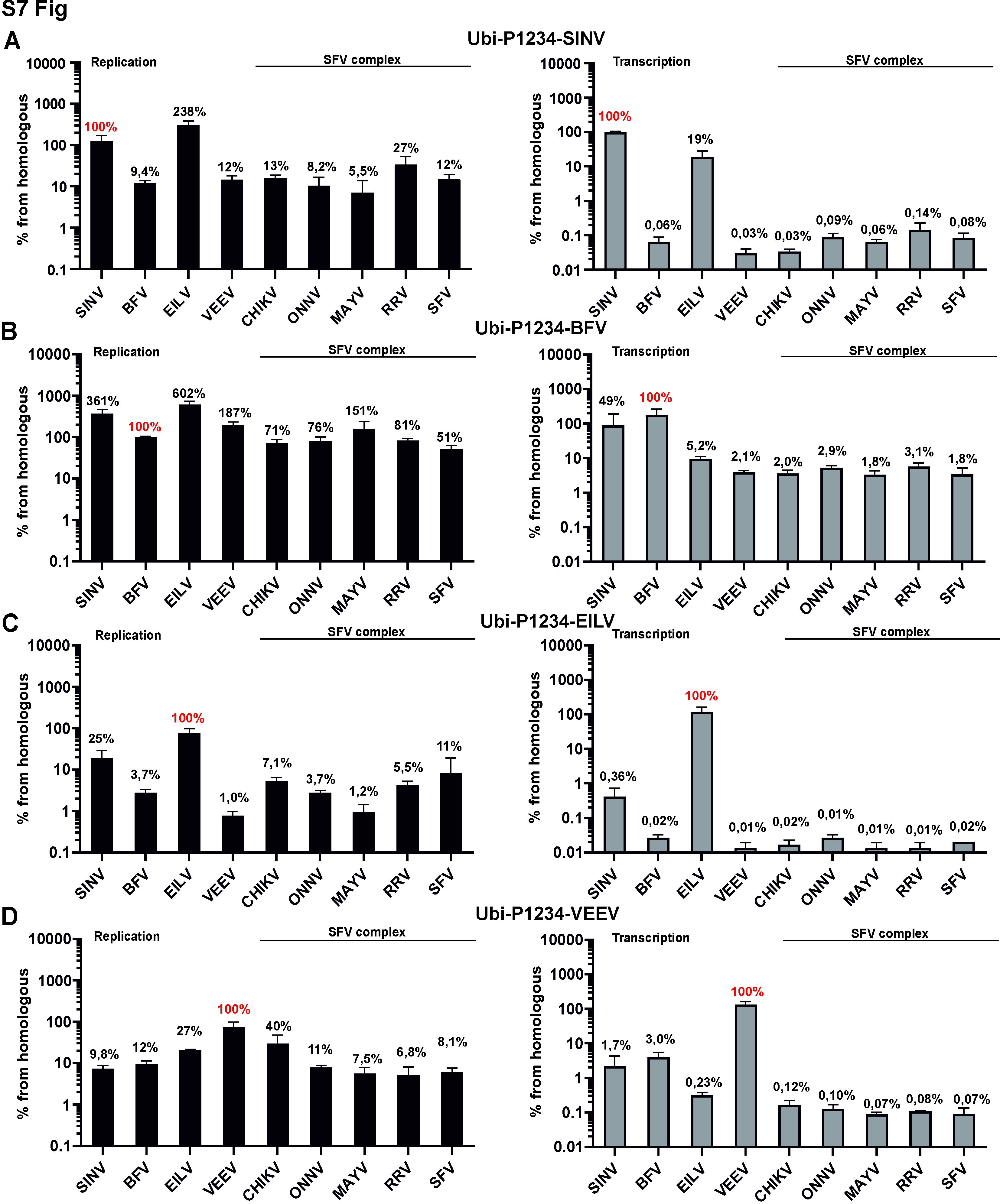

Supplement: S7 Fig — Comparison of capacities of replicases from outgroup alphaviruses to replicate (left) and transcribe (right) different template RNAs in Aedes albopictus C6/36 cells. Data is replotted from Figs 7 and 8. X-axis shows different templates; Y-axis shows percentage of activity of replicase on different templates; the activity on homologous template is taken as 100%. (TIF) [file ppat.1008825.s007.tif]
