# Supplementary material for: Cross-utilisation of template RNAs by alphavirus replicases
Source: PLoS Pathog. 2020 Sep 4;16(9):e1008825. doi: 10.1371/journal.ppat.1008825 (PMC7498090; doi:10.1371/journal.ppat.1008825)
Supplement: S8 Fig — In silico predicted UNAFOLD thermodynamic predictions for stable RNA structures within the SL3 region of SINV (S-S-S), CHIKV (C-C-C), SΔT-S-S, cT/14ss-S-S, cT/TATT/ss-S-S and a range of divergent alphaviruses. (PDF) [file ppat.1008825.s008.pdf]

S8 Fig

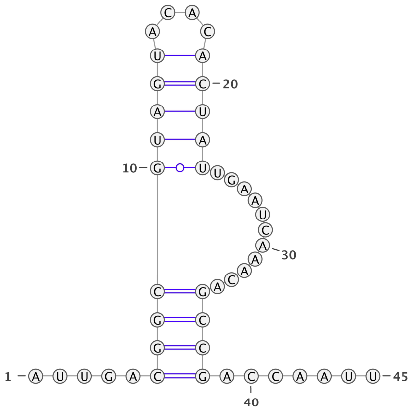

SINV (S-S-S)
- 28.9 kJ/mol

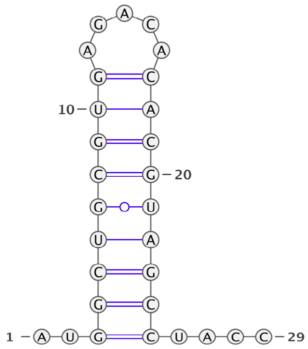

CHIKV (C-C-C)
- 65.7 kJ/mol

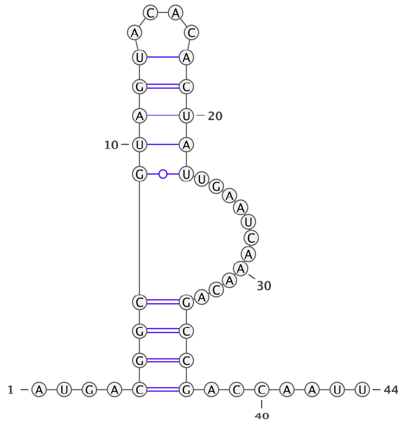

S$^{\Delta T}$-S-S
- 28.9 kJ/mol

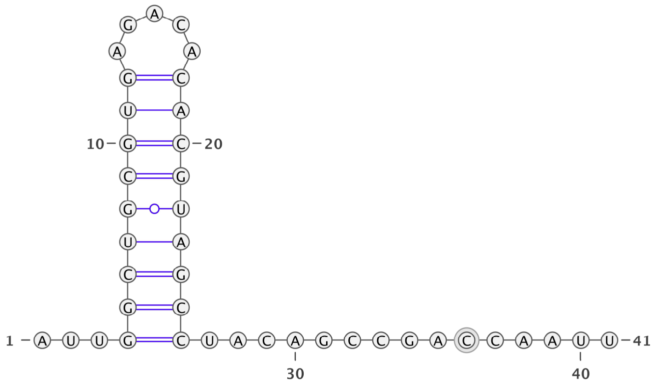

$c^{T/14}$ss-S-S
- 65.7 kJ/mol

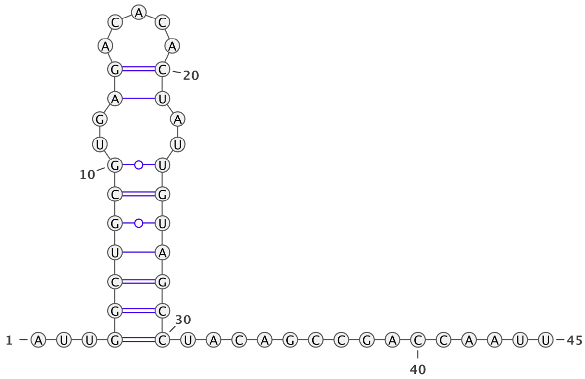

$c^{T/TATT/14}$ss-S-S
- 42.3 kJ/mol

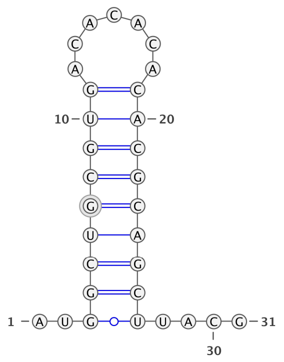

ONNV
- 61.9 kJ/mol

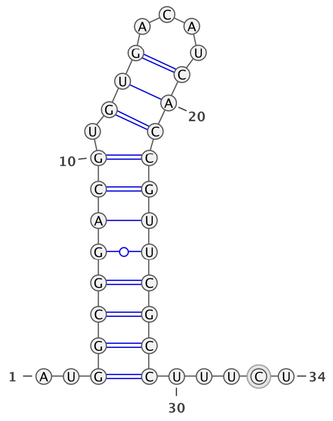

RRV
- 75.3 kJ/mol

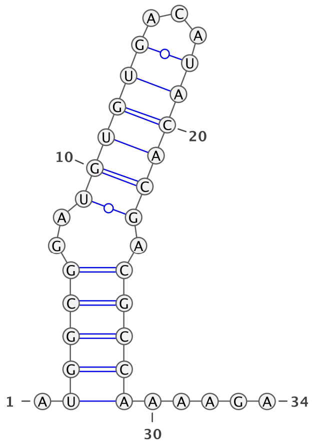

SFV
- 53.1 kJ/mol

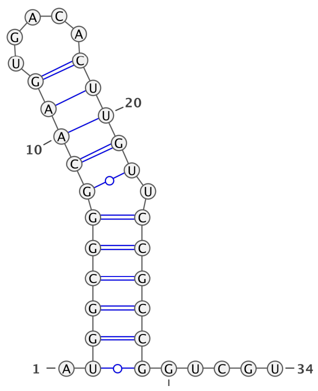

MAYV
- 66.9 kJ/mol

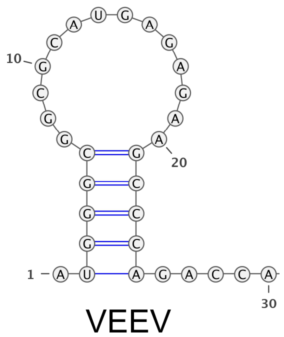

VEEV
- 33.5 kJ/mol

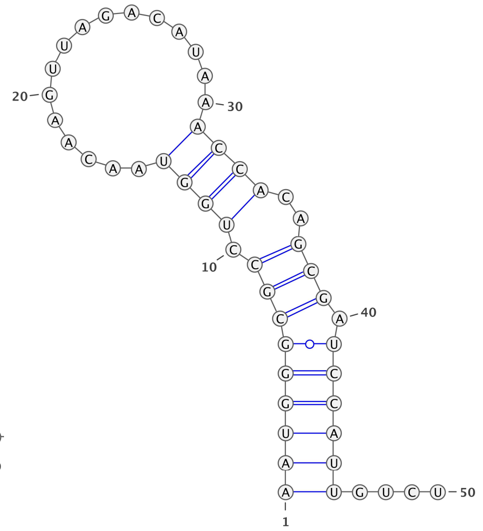

BFV
- 50.0 kJ/mol

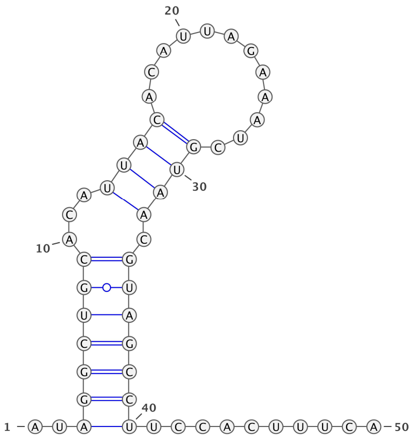

EILV_37^{o}C
- 43.9 kJ/mol

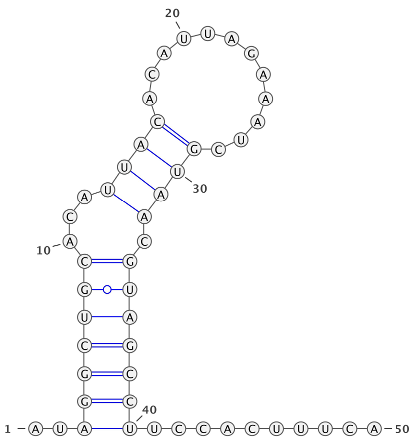

EILV_28^{o}C
- 54.9 kJ/mol
